# Adenoviral vector with shield and adapter increases tumor specificity and escapes liver and immune control

**DOI:** 10.1038/s41467-017-02707-6

**Published:** 2018-01-31

**Authors:** Markus Schmid, Patrick Ernst, Annemarie Honegger, Maarit Suomalainen, Martina Zimmermann, Lukas Braun, Sarah Stauffer, Cristian Thom, Birgit Dreier, Matthias Eibauer, Anja Kipar, Viola Vogel, Urs F. Greber, Ohad Medalia, Andreas Plückthun

**Affiliations:** 1 Department of Biochemistry, University of Zurich, Winterthurerstr, 190, 8057 Zurich, Switzerland; 2 Department of Molecular Life Science, University of Zurich, Winterthurerstr, 190, 8057 Zurich, Switzerland; 3 Laboratory of Applied Mechanobiology, Department of Health Sciences and Technology, ETH Zurich, 8093 Zurich, Switzerland; 4 Laboratory for Animal Model Pathology, Institute of Veterinary Pathology, Vetsuisse Faculty, University of Zurich, Winterthurerstrasse 268, 8057 Zurich, Switzerland; 5 Department of Life Sciences and the National Institute for Biotechnology in the Negev, Ben-Gurion University, Beer-Sheva, 84105 Israel

## Abstract

Most systemic viral gene therapies have been limited by sequestration and degradation of virions, innate and adaptive immunity, and silencing of therapeutic genes within the target cells. Here we engineer a high-affinity protein coat, shielding the most commonly used vector in clinical gene therapy, human adenovirus type 5. Using electron microscopy and crystallography we demonstrate a massive coverage of the virion surface through the hexon-shielding scFv fragment, trimerized to exploit the hexon symmetry and gain avidity. The shield reduces virion clearance in the liver. When the shielded particles are equipped with adaptor proteins, the virions deliver their payload genes into human cancer cells expressing HER2 or EGFR. The combination of shield and adapter also increases viral gene delivery to xenografted tumors in vivo, reduces liver off-targeting and immune neutralization. Our study highlights the power of protein engineering for viral vectors overcoming the challenges of local and systemic viral gene therapies.

## Abstract (web summary)

Viral gene therapy can be limited by the efficacy of virion sequestration, immune responses and the silencing of genetic payloads. Here the authors engineer an advenovirus protein coat which shields the virion from the immune system while targeting cancer cells.

## Introduction

Recent innovations in gene editing technologies and therapeutic benefits in clinical trials with protein therapeutics have brought the idea of viral gene therapy back to center stage^1^. This is highlighted by the approval of the first gene therapy in the United States and Europe^2^, an adeno-associated virus (AAV). Adenoviruses (AdVs) are the most widely used vectors in clinical gene therapy trials^3,4^. There are over 65 different human AdV types known, including adenovirus 5 from the species C (HAdV5), which infects respiratory epithelial cells and is normally well controlled by innate and adaptive immunity in immune-competent individuals^5^. HAdV5 is the best characterized adenovirus and a promising vector for gene delivery for the treatment of human diseases, such as cancer or germline defects^6^. HAdV5-based vectors are powerful for viral gene therapy since they have high transduction efficacy of non-dividing and dividing cells and can be readily produced in large scale and in clinical grade quality^7^. Importantly, the adenoviral genome remains episomal in transduced cells, which provides a safety margin over integrating vectors such as lentiviruses^8^. Recently, the development of ‘gutless’ AdVs, which are devoid of viral genes and thus extend safety margins even further, has enabled the utilization of up to 35 kilobase pairs (kb) for transgene expression, which exceeds the capacity of AAV vectors by one order of magnitude^9^. This will allow the delivery of multiple payload genes at once, and potentially the secretion of a cocktail of therapeutic proteins upon delivery of the non-oncolytic vector to a tumor^10^, without vector replication and thus with enhanced safety.

Advances in vector development have significantly improved adenovirus efficacy, yet the targeting of the vectors to the tissue of interest by systemic delivery remains a challenge. One hurdle is the strong liver tropism of HAdV5 upon intravenous administration, minimizing the delivery efficacy to a tumor^11^. Other limitations are the preexisting humoral immunity against the vector and the innate and adaptive immune responses triggered by the vector^12^. Antibody neutralization of the virion can occur at the level of virus entry into cells, for example, by blocking receptor binding or endosomal escape of the virion^13,14^. For AdVs, antibody-dependent intracellular neutralization (ADIN) constitutes another pathway of neutralization, which targets the virion to proteasomal degradation^15,16^. The innate immune system inactivates virions through preexisting immunoglobulin M (IgM) antibodies and the complement system^17,18^. Binding of coagulation factor X (FX) precludes the binding of those antibodies to the virion^19^. FX binds to the central cavity of a hexon trimer, thereby creating a shield around the capsid^20^. On the other hand, this FX shield enhances the transduction of hepatocytes and other cells, likely through virion attachment to heparan sulfate proteoglycans^21^. Furthermore, if FX-shielded virions enter the cytosol, FX can act as a pathogen-associated molecular pattern (PAMP) and trigger innate immunity against the infected cells, which in turn may reduce the lifetime of the transduced cells^22^.

So far, most efforts to shield virions from these undesired interactions have focused on polymers, like polyethylene glycol (PEG), *N*-(2-hydroxypropyl)methacrylamide or poly(amidoamine), sometimes fused to cationic moieties for simple electrostatic binding to the particles. However, the heterogeneous size of the polymers, the non-covalent ionic linkage to the capsid and inherent chemical features of the polymers altered the infectivity of the virions in unpredictable manners, and largely reduced the transduction efficiency^23,24^. Likewise, shielding attempts with engineered protein coats showed promising results in vitro, but were ineffective in vivo^25^.

Besides overcoming detargeting, another challenge for viral tumor gene therapy is the targeting of the vector to the cancer cells. Infection of epithelial cells requires interactions of the vertex-associated fiber and penton base proteins with their cognate receptors, the *C*oxsackievirus and *A*denovirus *R*eceptor (CAR) and αV integrins^26,27^. These receptor interactions firmly tether the virion to the cell surface and trigger the first steps of the virion uncoating program^28–31^. Virion binding to CAR and integrin receptors triggers endocytosis and escape to the cytosol from non-acidic early endosomes^32^.

Virus retargeting to cancer cell epitopes has been achieved by genetic engineering of the fibers^32^, or modular adapter systems^33,34^. The latter has allowed retargeting of HAdV5 to several cancer biomarkers, such as human epidermal growth factor receptor 2 (HER2) or epidermal growth factor receptor (EGFR)^34^. The adapter consists of a central fiber knob-binding Designed Ankyrin Repeat Protein (DARPin) fused on one side to the phage protein SHP, which mediates extremely stable trimerization and thus formation of a highly stable complex of the DARPins and fiber knob without measureable off-rate over 10 days^34^. The other end of the fiber knob-binding DARPin is connected via a flexible linker to an exchangeable retargeting module that can bind cell surface receptors. In addition to allowing retargeting to a tumor-specific receptor, the adapter also blocks large areas of the knob surface, which precludes knob binding to CAR and thus reduces transduction of CAR-positive cells.

In order to engineer a protecting function of a coat but without the liabilities of FX, we designed in this study a ‘stealth’ layer based on a hexon-binding humanized single-chain antibody variable fragment (scFv) derived from the murine monoclonal antibody (mAb) 9C12^35^. The shield has low pM affinity and extensively covers the surface of HAdV5, as indicated by single-particle electron microscopy analyses and an atomic-resolution crystal structure of the hexon–scFv complex. We demonstrate maintained in vitro and in vivo transduction efficacy of a novel engineered FX-ablated non-replicative HAdV5 with a designed protein shield. By implementing both retargeting and shielding strategies, we achieved viral gene delivery to two different tumor models overexpressing HER2 or EGFR in vivo, reduced the off-targeting to the liver and increased the tumor-to-liver-ratio of the expressed transgene by a factor of about 2500 compared to naive HAdV5 vectors.

## Results

### Adapter-mediated retargeting of capsid-engineered virions

**Fig. 1:**
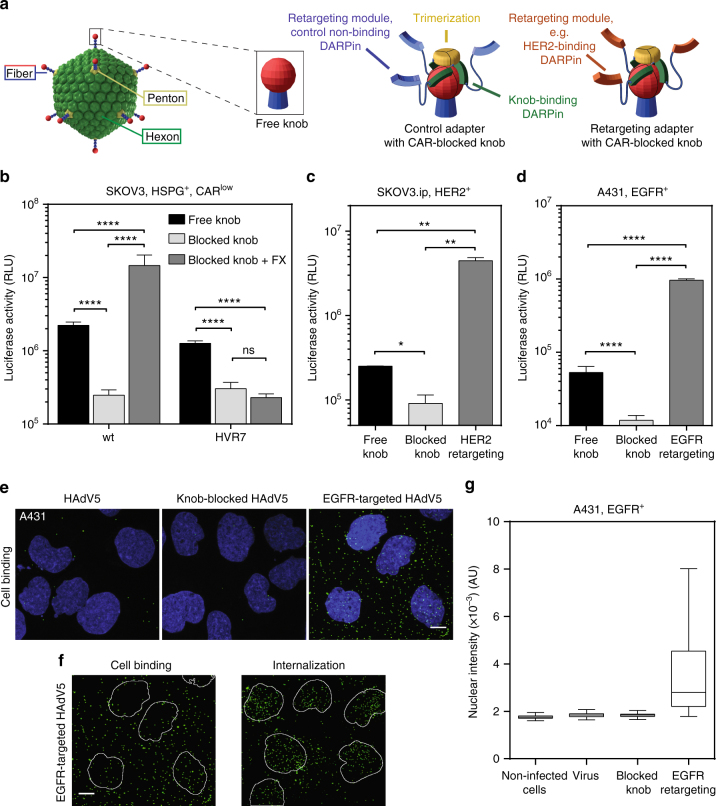
Retargeting of FX-binding-ablated HAdV5^HVR7^ to HER2^+^ and EGFR^+^ tumor cells. **a** Overview of the knob-adapter complexes. Knob-binding DARPins are trimerized through SHP and bind the knob in a quasi-covalent manner^34^. The retargeting module (orange), a target-specific DARPin, allows binding of tumor biomarkers like HER2 or EGFR. Non-targeting (control) DARPins are shown in blue. **b** Hexon-engineered HAdV5^HVR7^ infects CAR-expressing SKOV3 cells with similar efficiency as the wt virus, but addition of FX boosted only wt transduction, since HAdV5^HVR7^does not bind FX. RLU, relative light units; HSPG, heparan sulfate proteoglycan. **c**,** d** Knob binding of adapter decreased HAdV5^HVR7^ viral gene delivery to tumor cells in both **c** SKOV3.ip and **d** A431 cells through blocking of CAR interaction. Fusion of a HER2- or EGFR-binding DARPin to the adapter resulted in a ×50, or ×80 increase of tumor cell transduction, respectively. Shown are sample means ± SD from biological replicates ((**b**) *n*=4; (**c**) *n* = 2; (**d**) *n* = 4), two-way ANOVA of log-transformed data, *P < 0.05, **P < 0.01, ****P < 0.0001. **e**,** f** Analysis of binding and internalization of Alexa-Fluor 488-labeled HAdV5^wt^ to A431 cells. Viruses were bound to cells at +4 °C for 1 h and were fixed immediately or after subsequent 1 h of incubation at 37 °C. Images shown represent maximum projections of individual confocal stacks. Nuclei (DAPI stain) are blue and virus particles green. **e** The EGFR-retargeted virus showed increased cell binding in comparison to wt or fiber knob-blocked viruses and bind all over the cell. Pictures from the confocal microscopy are maximal projections of the cells, not slices through one plane, as explained in further detail in Supplementary Fig. 1. Scale bar = 10 µm. **f** At 4 °C (left), cell binding occurs, while at 37 °C internalization of EGFR-mediated HAdV5^wt^ results in nuclear trafficking of the particles. Nuclei are shown as outlines. Scale bar = 10 µm.** g** Retargeting of fiber-blocked virus to EGFR increases transduction of A431 tumor cells. A431 cells were imaged by automated fluorescence microscopy. The mean GFP intensity over a DAPI mask was quantified in single cells. In the box-and-whisker plots, center lines show the mean; box limits the 25^th^ and 75^th^ percentiles; whiskers according to Tukey. For each condition between 4 and 9 × 10^3^ cells were analyzed. AU, arbitrary units

Biodistribution of therapeutic viruses within the body is subject to manifold interactions of the virions with tissue-resident and circulating blood cells and lymphoid tissue, as well as soluble factors like blood coagulation FX. FX endangers the success of an adenoviral gene vector as it activates the innate immunity in the transduced cells^22^ and mediates liver uptake^36^. Therefore, we ablated binding of FX to HAdV5 by mutating the FX binding site on the hexon as recently reported^37^. Four mutations in the hypervariable region 7 (HVR7) of the hexon protein were introduced (I421G, T423N, E424S and L426Y), resulting in HAdV5^HVR7^. To enhance the targeting of virions to tumor cells of interest, and reduce virion attachment to endogenous fiber receptors, we made use of the recently developed adapter, which binds to and blocks the viral fiber knob (Fig. 1a)^34^.

To test whether FX binding was truly ablated, we infected SKOV3 cells, which express heparin sulfate proteoglycans on the cell surface^38^, with either HAdV5^wt^ or FX-binding-ablated HAdV5^HVR7^. With the adapter^34^ tightly bound to the fiber knob, CAR-mediated luciferase transgene expression should be reduced, as only FX-mediated entry was expected to take place. Indeed, blocking of the fiber knob itself reduced transduction of SKOV3 cells by both HAdV5^wt^ and HAdV5^HVR7^ (Fig. 1b), and in the presence of FX, transgene expression of the fiber knob-blocked HAdV5^wt^ was increased by 100-fold. In contrast, FX had essentially no effect on the transduction of HAdV5^HVR7^, indicating effective inhibition of FX binding.

We further applied the adapter retargeting strategy^34^ to the FX-ablated HAdV5^HVR7^. Depending on the retargeting module, the virus could be retargeted to cancer cells such as A431, expressing EGFR, or SKOV3.ip cells, expressing HER2 (Fig. 1c, d). Confocal microscopy imaging of A431 cells infected with Alexa-Fluor 488-labeled HAdV5^wt^ indicated that virions with normal or adapter-blocked fiber knobs bound inefficiently to these cells (Fig. 1e). In contrast, an EGFR-retargeting adapter led to significant increase of virion binding to cells. Importantly, the modification of the viral entry mechanism by targeting a non-native receptor did not ablate the cell entry and nuclear trafficking of the virus (Fig. 1f and Supplementary Fig. 1). EGFR retargeting enhanced cell binding and subsequently increased the number of internalized particles, which in turn also resulted in higher transgene expression (Fig. 1g). In conclusion, viral gene delivery by FX-ablated HAdV5^HVR7^ to cancer cell lines can be strongly enhanced with the fiber knob adapter strategy that targets tumor surface biomarkers.

### Retargeting improves gene delivery to xenograft tumors

Having shown the potential of FX-ablated retargeted viruses in vitro, we were interested in analyzing their behavior in vivo. Today, most clinical trials of virotherapy have used direct intratumoral delivery of the virion^39^, and thus we tested this approach first. To gain insight into the retargeting of HAdV5^HVR7^ in vivo we analyzed viral gene delivery in two subcutaneous xenograft models in immunodeficient *Rag1*^-/-^ mice (an immunodeficient strain that lacks mature T and B lymphocytes)^40^, thereby testing the performance of the DARPin adapter. Control experiments in an A431 cell culture indicated that there the adapter is stable in Rag1^-/-^ mice serum, since preincubation of adapter in serum had no significant impact on infection efficacy (Supplementary Fig. 2a).

**Fig. 2:**
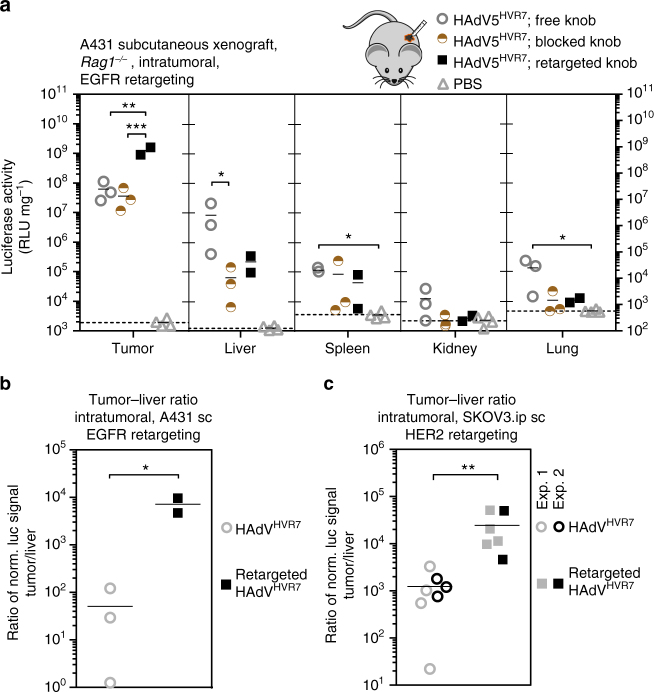
Retargeting of HAdV5^HVR7^ increases tumor-specific gene delivery after intratumoral injection. **a** 1.5 × 10^6^ HAdV5^HVR7^ particles were injected into subcutaneous EGFR^+^ A431 tumor xenografts in *Rag1*^-/-^ mice. Gene delivery was analyzed 48 hpi by luciferase activity, and the values obtained were normalized to total protein amount. The experiment was performed with randomized groups and blinded. Virus alone (free knob) showed significant transgene signal in all analyzed organs other than kidney. Gene delivery to the liver was reduced by blocking the fiber knob with the adapter (blocked knob). EGFR-specific retargeting adapter significantly increased tumor infection (retargeted knob). Background signals from control injections with PBS are indicated by dashed lines (mean, each symbol represents one organ, *n* = 2–3 mice per group. RLU, relative light units. One-way ANOVA of log-transformed data, *P < 0.05, **P < 0.01, ***P < 0.001). **b**, **c** The tumor-to-liver ratio was calculated for each individual mouse. The tumor-to-liver ratio was 50 for the unmodified virus and 7200 for virus retargeted to EGFR (**b**). In the case of HER2 xenografts, the values were 1200 and 24,600 for unmodified and HER2-retargeted virus, respectively (**c**). Sc, subcutaneous. Pooled data from repeated independent experiments were used for statistics, and individual experiments are indicated (mean, each symbol represents the ratio of an individual, two-sided, unpaired Welch’s *t*-test of log-transformed data, *P < 0.05, **P < 0.01)

Upon intratumoral administration into A431 tumor xenografts, an EGFR-specific retargeting adapter increased the payload delivery (luciferase) in the tumor by 20-fold, compared to HAdV5^HVR7^ with a free fiber knob, and by 34-fold compared to a blocked fiber knob virus (Fig. 2a). At the same time, the binding of the adapter decreased liver targeting by approximately 37-fold compared to the non-targeted HAdV5^HVR7^. In lung, spleen and kidney, the luciferase signal was lower than in the liver, and the signal was essentially at background levels in kidney and lung when blocking the fiber knob. In the context of viral therapeutic gene delivery, the ratio of expressed payload genes between tumor and liver is of high relevance due to potential off-target side effects of future payloads. In the A431 EGFR^+^ tumor model, intratumoral application of HAdV5^HVR7^ thus led to a tumor-to-liver ratio of 50, which was strongly increased to about 7200 by EGFR targeting and inhibition of the CAR uptake pathway, representing a 140-fold gain in specificity (Fig. 2b).

A HER2-overexpressing SKOV3.ip xenograft model in *Rag1*^-/-^ mice showed similar effects. Using the knob-binding adapters, liver targeting was significantly reduced by up to 30-fold (Supplementary Fig. 2b). With the HER2-specific retargeting module, the payload delivery to the tumor was increased by eightfold compared to the fiber knob-blocked virus upon intratumoral virus injection. The signals in kidney and lung were decreased by the adapters essentially to background levels. Also in the HER2^+^ tumor model, the tumor-to-liver ratio of the delivered viral payload of about 1200 was increased to about 24,600 by HER2-retargeting and fiber knob blocking, a 20-fold change of tumor selectivity (Fig. 2c). Thus, the application of a retargeting and CAR pathway-blocking adapter in combination with FX-ablated HAdV5^HVR7^ improved the localization upon intratumoral vector administration, being encouraging for future therapeutic strategies.

### EGFR retargeting enhances tumor cell-specific gene delivery

**Fig. 3:**
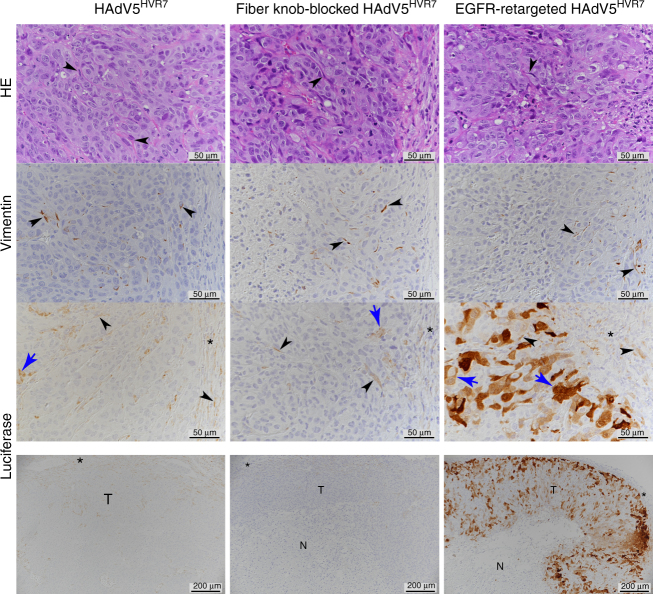
EGFR-retargeting results in gene delivery to A431 tumor cells after IT injection. In situ assessment of viral transgene expression in A431 xenograft tumors by immunohistology. Host fibroblasts are detected based on the expression of vimentin; luciferase staining reflects viral transgene expression. With both free virus and fiber knob-blocked virus, mostly fibroblasts (arrowheads) express the transgene luciferase, both in the tumor and in the surrounding stroma (asterisks). Luciferase-positive neoplastic cells (blue arrows) occur only rarely. In contrast, EGFR-retargeted virus is mainly found in viable tumor cells (blue arrows), although stromal fibroblasts are also luciferase positive (arrowheads). Analysis of tumor sections was conducted in a blinded fashion, and tumor cells were confirmed by their EGFR expression. Scale bar = 50 µm. The lower row provides an overview of the tumor after luciferase staining (N necrotic tumor tissue, T viable tumor tissue); here scale bar = 200 µm

Next, we evaluated the cellular specificity of viral gene delivery in A431 tumor xenografts in more detail. Immunohistology for luciferase transgene expression revealed that the untargeted HAdV5^HVR7^ virus almost exclusively infects murine fibroblasts or fibrocytes after intratumoral administration (Fig. 3). Only an occasional luciferase-positive tumor cell was observed. The fiber knob-blocked virus showed a similar result. In contrast, virus with the EGFR-retargeting adapter resulted in luciferase expression in tumor cells, which were mainly observed as large patches. Nonetheless, also the retargeted virus transduced fibroblasts in the tumor-surrounding stroma. These results show that, even after intratumoral injection, gene delivery with unmodified HAdV5^HVR7^ is limited to murine stromal cells. The present data reveal the necessity of a tumor-specific targeting adapter module to infect the A431 tumor cells and demonstrate its functionality in vivo. It is worth noting that only in the case of the unmodified HAdV5^HVR7^, but not for the adapter-bound virions, luciferase-positive hepatocytes could be detected in the liver (Supplementary Fig. 3a). This is in accordance with the observed reduction of liver targeting in the xenograft (Fig. 2a).

### Adapter reduces liver tropism in systemic injections

**Fig. 4:**
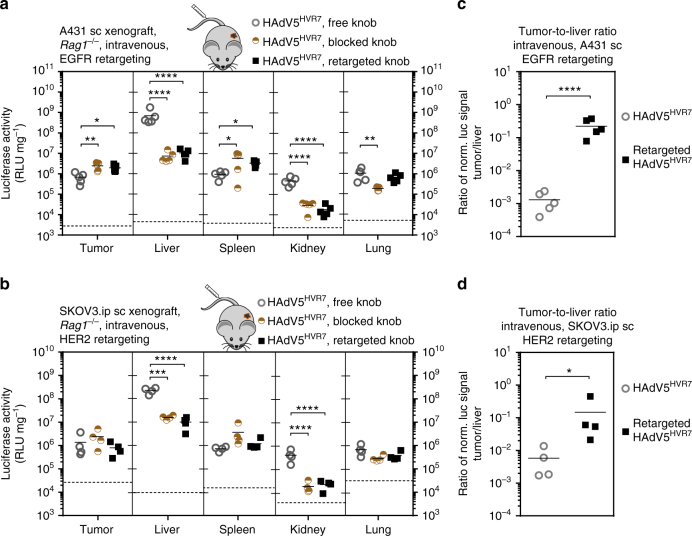
Adapter reduces off-targeting and increases tumor–liver ratio after intravenous (IV) injection. **a**,** b** 3 × 10^6^ HAdV5^HVR7^ particles were injected in the tail vein of *Rag1*^-/-^mice, bearing either EGFR- or HER2-overexpressing subcutaneous tumors. Gene delivery was analyzed 48 hpi by luciferase activity, normalized to total protein amount. **a** More than 99% of the transgene activity was located in the liver after IV application of the virus alone. The blocking of the fiber knob by either adapter significantly decreased the gene delivery to liver, kidney and lung. Either knob-binding adapter increased the gene delivery to the tumor and the spleen. Background signals from control injections with PBS are indicated by dashed lines (each symbol represents one organ, *n*=5, two-way ANOVA of log-transformed data, *P < 0.05, **P < 0.01, ***P < 0.001, ****P < 0.0001). **b** Intravenous injection of blocked, non-targeted HAdV5^HVR7^, and HER2-targeted HAdV5^HVR7^ resulted in strong reduction of liver and kidney transduction. A HER2 adapter-mediated targeting of the tumor was absent, since the luciferase activity was not higher than for the blocked virus. Background signals from control injections with PBS are indicated by dashed lines (each symbol represents one organ, *n* = 4, two-way ANOVA of log-transformed data, ***P < 0.001, ***P < 0.0001). **c** The ratio of transgene activity between tumor and liver was increased by the EGFR-retargeting adapter by a factor of 170 (each symbol represents the ratio of an individual mouse, two-sided, unpaired Welch’s *t*-test of log-transformed data, ****P < 0.0001). **d** The HER2-specific adapter increased the tumor–liver ratio in the SKOV3.ip xenograft by a factor of 25 (each symbol represents the ratio of an individual mouse, two-sided, unpaired Welch’s *t*-test of log-transformed data, *P < 0.05)

Intratumoral administration of therapeutics has limited benefit for the patient with disseminated tumors, as it is not applicable to poorly accessible tumors. We thus explored systemic delivery of HAdV5^HVR7^, where native particles are rapidly scavenged by the liver^41^. We investigated the impact of the fiber knob-binding adapters in EGFR^+^ (A431) and HER2^+^ (SKOV3.ip) subcutaneous xenograft models with HAdV5^HVR7^ upon intravenous injection. The retargeting adapter reduced the viral liver tropism around 70- and 20-fold in the A431 and SKOV3.ip xenograft models, respectively (Fig. 4a, b). Similar results were obtained with viruses containing the blocking adapter. This suggests that the knob is playing an active role in liver tropism. Surprisingly, blocking of the fiber knob with the adapter also strongly reduced off-targeting to the kidney. In contrast, the signals in spleen and lung remained unchanged. However, no increase in tumor targeting by the retargeting adapter was detected after intravenous administration, compared with the knob-blocking adapter, although the tumor-to-liver ratio of payload expression was significantly increased by a factor of around 170 in the EGFR^+^ tumor model and by 25-fold in the HER2^+^ tumor model (Fig. 4c, d), compared to the naked HAdV5^HVR7^.

### Design of a capsid-binding shield based on a humanized scFv

**Fig. 5:**
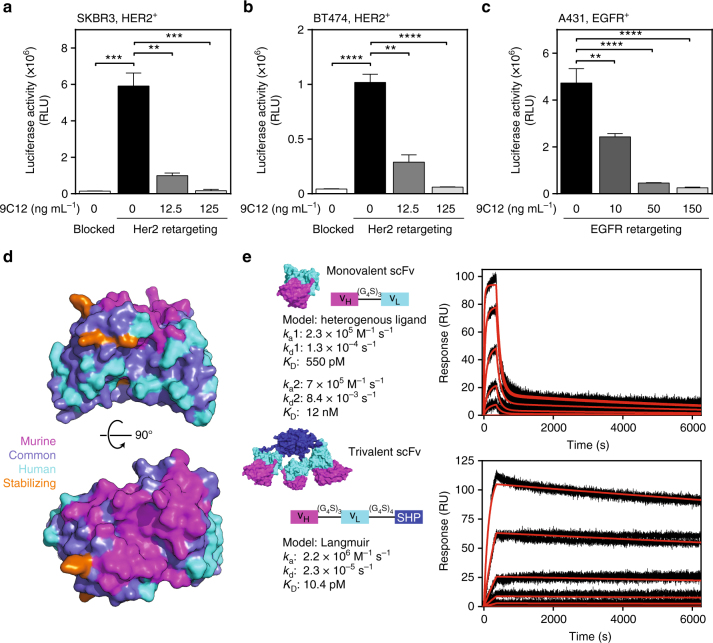
Design of a hexon-binding trimerized scFv with low picomolar affinity to hexon. **a**–**c** HAdV5^HVR7^ retargeted by an adapter to HER2- or EGFR-expressing tumor cells are still susceptible to ADIN by the mAb 9C12 in a concentration-dependent manner. Blocked denotes the blocking adapter without targeting function (mean ± SD, *n*=2-3, one-way ANOVA of logarithmic data, *P < 0.05, **P < 0.01, ***P < 0.001, ****P < 0.0001). Note that the blocked control for **c** is shown in Fig. 1d. **d** Humanization of a scFv by CDR grafting on a stable human framework. Model of scFv colored by sequence origin. **e** For trimerization, the scFv was fused to the phage SHP protein^43^ and this resulted in stable trimers in solution. The scFv consists of a heavy chain followed by a light chain connected with a glycine–serine linker. Affinity of monovalent and trivalent scFv measured by SPR with immobilized hexon. Monovalent scFv was injected at five different concentrations (1 nM, 3.16 nM, 10 nM, 31.6 nM, and 100 nM). A heterogenous ligand model resulted in two *K*_D_s of 12 nM and 550 pM. The trivalent scFv was injected in concentrations of 31.6 pM, 100 pM, 316 pM, 1 nM, and 3.16 nM. Fitting the data with a Langmuir model resulted in an affinity of 10 pM

We next analyzed viral payload expression in different tumor cell lines in the presence of the hexon-specific antibody 9C12^35^. As expected, immunoglobulin G-mediated ADIN-based neutralization prevented viral payload delivery in SKBR3, BT474 and A431 cells, when cell entry occurred via an adapter targeting the virion to EGFR or HER2 (Fig. 5a–c). To overcome these limitations, and to protect the virion from undesired interactions with host factors, we designed an artificial protein-based shield around the hexon shell protein of the virion.

The construction of a humanized scFv provided the basic module of the protein shield (Supplementary Fig. 4a). A CDR (complementarity-determining region) graft from the murine hexon-binding mAb 9C12 onto a human scFv framework led to a stable, monomeric scFv (Fig. 5d and Supplementary Fig. 4b)^35,42^. To increase the affinity of the shielding scFv to multimers of hexons as present in the virion capsid, we constructed a trimeric scFv by fusing a highly stable trimerization domain, SHP of lambdoid phage 21^43^, to the carboxy-terminus of the scFv (Supplementary Fig. 4c, d). This strategy exploits the various threefold symmetry axes present on the triangular faces of the icosahedral shell. Trimerization led to an increase in affinity from low nM of the monovalent scFv to 10 pM of the trivalent scFv to immobilized hexons (Fig. 5e). In contrast to the monovalent scFv, the trivalent scFv blocked binding of a bivalent mAb to both purified hexon and virions (Supplementary Fig. 5a, b). We therefore conclude that the trivalent scFv provides a high-affinity shield on the virion surface, most likely through avidity effects.

### Shield covers virion by binding the domains of a hexon trimer

**Fig. 6:**
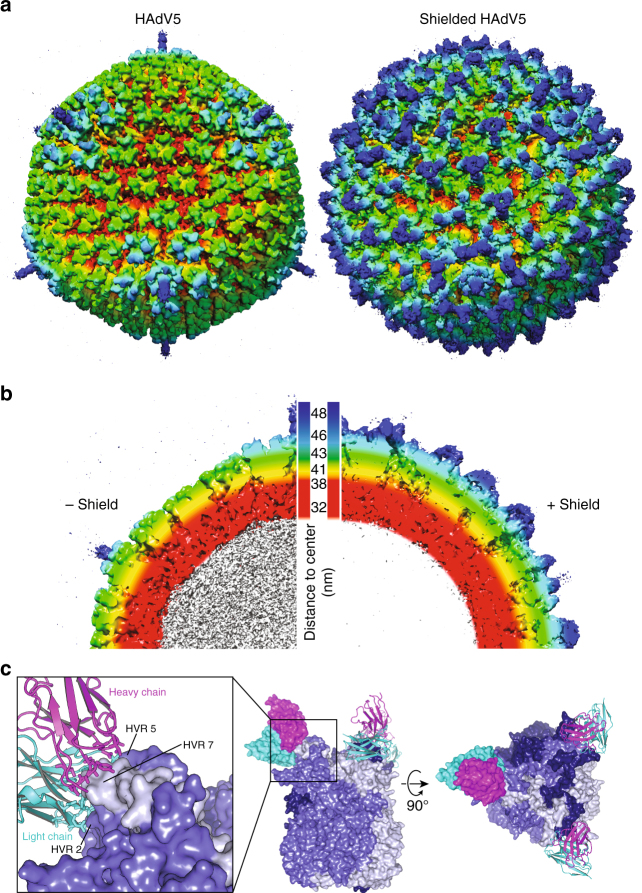
EM structure of shielded virus and crystal structure of hexon–scFv complex. **a**,** b** Comparison of EM structure of naked and shielded HAdV5. Color reflects distance to the core (white: <32 nm, red: 32–38 nm, yellow 41 nm, green 43 nm, cyan 46 nm, blue 48 nm). Trivalent shield proteins (green–blue) bind all over the capsid, resulting in a dense cover of viral capsid proteins (red–light green). **c** High-resolution crystal structure of scFv–hexon complex elucidates the atomic interactions. Both heavy (magenta) and light (cyan) chain of the scFv bind to the tower of a hexon monomer (three different shades of blue, one scFv displayed as surface, others as cartoon), formed mainly by HVR2 and HVR7. The structure also shows few interactions with HVR5. Importantly, all three epitopes in the trimeric hexon were occupied with three scFvs

To evaluate the arrangement of the shield on the icosahedral capsid, the structure of the complex between HAdV5^HVR7^ and the trimeric scFv shield was determined by single-particle cryo-electron microscopy to a resolution of 7.4 Å (Supplementary Fig. 6). A three-dimensional (3D) reconstruction revealed additional density all around the capsid (Fig. 6a). In the naked capsid, the deep canyons between the inner capsid shell (red) and the trimeric hexons (yellow and green) are clearly visible by applying a color gradient indicating the distance to the center of the virion (Fig. 6b). The shield expanded the diameter of the hybrid particle by approximately 10 nm from 88 to 98 nm (Supplementary Fig. 7a), thereby masking the icosahedral shape of the capsid and yielding a rather spherical object.

To explore atomic details of the hybrid particle we also determined the X-ray crystal structure of the scFv–hexon complex (Fig. 6c and Supplementary Fig. 8). The high-resolution structure of the hexon and a 9C12 antibody fragment unveiled the complex interaction network between the heavy and the light chains of the scFv and different domains of a hexon trimer, especially HVRs 2, 5 and 7. In order to assess possible differences between the crystal and the in situ structure of the scFv–hexon complex, we performed molecular dynamics flexible fitting (MDFF) with the electron microscopy (EM) map of the shielded capsid. During a 1 ns MDFF run we observed only a very minor increase of the cross-correlation between map and structure from an initial value of 0.87 to a plateau value 0.90. This was mostly caused by a slight reorientation of the scFv on the hexon that did not affect epitope binding (Supplementary Fig. 7b). The high similarity between the two structures was also reflected by a root-mean-square deviation of only 1.5 Å (Supplementary Fig. 7c). This is further evidence for the validity of the structure of the complex and underlines the high stability of the trimeric shield and the hexon trimer.

**Fig. 7:**
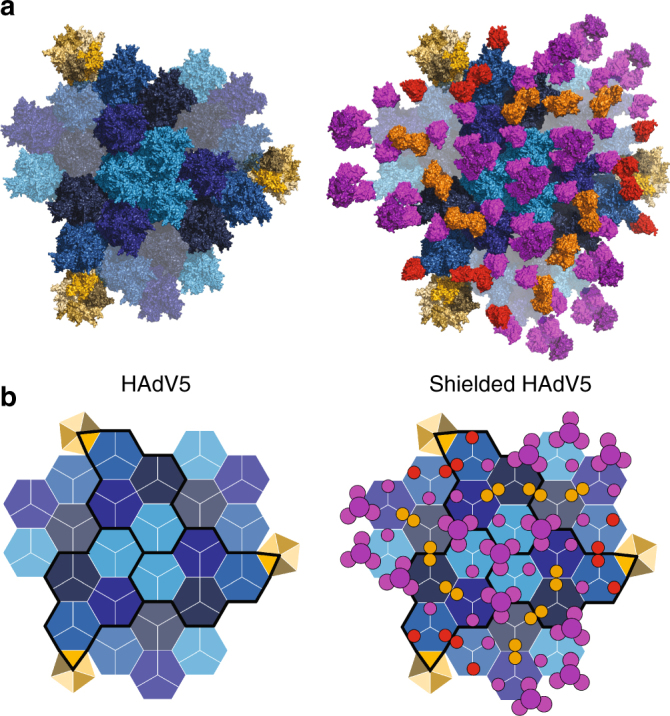
Projection of crystal structure explains binding stoichiometry on viral surface. **a** Structural and **b** schematic representation of shield occupancy and stoichiometry on viral capsid. One triangular capsid face is formed by three asymmetric units (AU, black outlines, consisting each of one pentameric subunit (yellow) and four hexon trimers (different shades of blue)). Hexons adjacent to the black outline belong to the neighboring facet. For the shielded surface, all scFvs were placed onto the hexons according to the crystal structure. At the threefold symmetry axes between neighboring hexons, a complete trivalent scFv-SHP could bind. The projection of the structure predicted a clash if the tower of two neighboring hexons face each other (orange scFvs or circles). At the vertices close to the penton, the surface is bent, which results in only a minor clash (red scFvs and circles)

To analyze the stoichiometry and the surface occupancy of the shield on the viral capsid, we projected the complex structure onto 6 asymmetric units (AUs) of one triangular facet of the icosahedral capsid (Fig. 7a, b). Binding of the scFv is influenced by the symmetrical arrangement of the neighboring hexons: each icosahedral facet comprises 72 epitopes of mAb 9C12; at 42 epitopes, the scFvs bind without any clashes. Especially around the threefold axes between hexons, the binding was sufficient for the flexibly linked SHP to be resolved in the EM structure. At 18 epitopes two towers of neighboring hexons are facing each other, resulting in a clash of the respective scFvs. At the 12 epitopes around the pental vertex, the viral surface is slightly tilted, which results in less clash volume. As a result, more additional shield density was observed in the EM structure at these positions.

### Shield keeps virus infectivity and inhibits neutralizing Abs

**Fig. 8:**
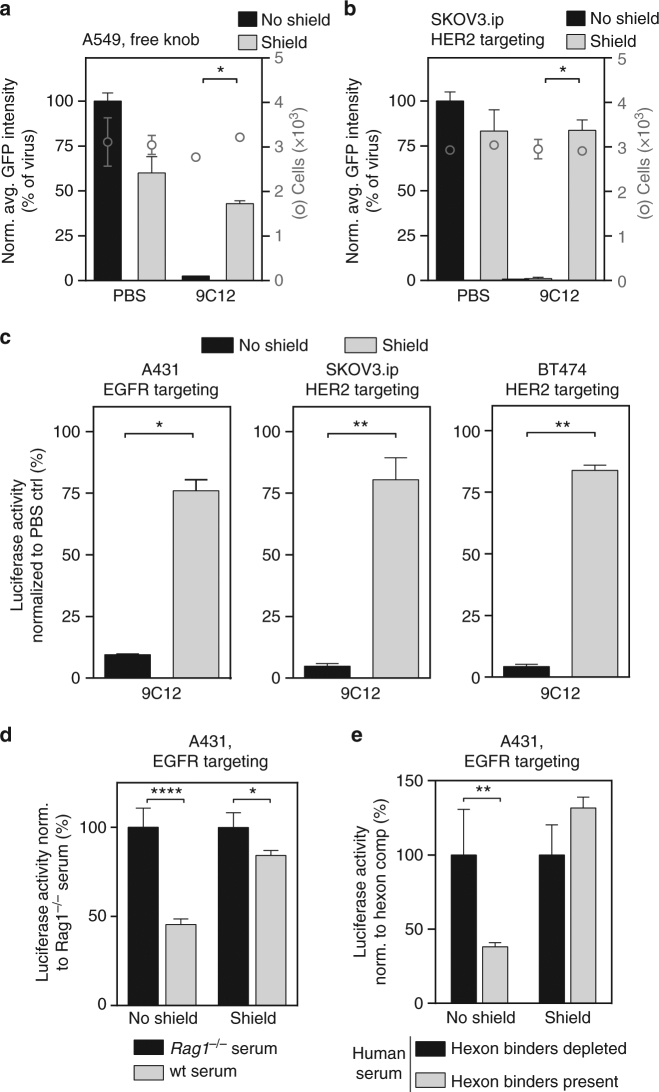
Shielding of HAdV5^HVR7^ reduces neutralization through immune system. **a**,** b** Single-cell analysis of GFP reporter by HT-microscopy indicated more than 95% reduction of gene delivery with HAdV5 in the presence of anti-hexon mAb 9C12. If virus was shielded by the trivalent scFv, HAdV5 infection of **a** A549 cells or infection of **b** SKOV3.ip cells was not significantly affected by the 9C12 antibody (mean ± SD, two-sided, unpaired Welch’s *t*-test, *n* = 2, *P < 0.05). For each condition, around 3000 cells were analyzed. **c** Antibody-dependent neutralization of HAdV5^HVR7^ can be blocked by the trivalent shield as shown by infection assays in A431 (EGFR targeting), SKOV3.ip (HER2-targeting), or BT474 (HER2-targeting) cells (mean ± SD, two-sided, unpaired Welch’s *t*-test, *n*=2-3, *P < 0.05, **P < 0.01). **d** Incubation of virus with wt mouse serum reduced viral gene delivery to 45% compared to *Rag1*^-/-^ serum (set to 100%), while the shielded virus was only slightly affected by the wt serum (84%) (mean normalized to *Rag1*^-/-^ ctrl ± SD, two-way ANOVA of normalized data with post hoc Bonferoni, *n* = 4, *P < 0.05, ****P < 0.0001). **e** Analysis of whether neutralization by human serum is due to hexon-specific antibodies. Transduction was performed with or without soluble hexon as competitor for such antibodies; presence of 0.88 µM hexon was set to 100%. Unshielded HAdV5^HVR7^ is neutralized by human serum in the presence of hexon binders, i.e., when they are not removed, but the transduction of the shielded virus is similar in the presence or absence of hexon-specific binders in human serum (mean normalized to hexon-depleted condition ± SD, two-way ANOVA of normalized data, *n* = 4, **P < 0.01)

We next investigated whether the artificial trimeric protein shield prevented virus neutralization. A single-cell transduction analysis of A549 cells using high-throughput microscopy showed an efficient reduction in the infectivity of the unshielded HAdV5^HVR7^ when mAb 9C12 was added, consistent with the reported ADIN^16^. This neutralization was significantly blocked by the shield (Fig. 8a). We next analyzed the effect of the shield in the context of the retargeting adapter. Single-cell transduction analysis of SKOV3.ip cells with HER2-retargeted virus confirmed the efficacy of the shield against antibody-mediated neutralization (Fig. 8b). It has been shown that this ADIN-mediated viral degradation is proteasome dependent^16^ for HAdV5^wt^ using the CAR pathway. Inhibition of the proteasome with MG132 in HER2-overexpressing SKBR3 cells diminished ADIN-mediated viral degradation, indicating proteasome dependency for HER2-retargeted virions as well (Supplementary Fig. 9a)^16^. The retargeting of a shielded virus was further studied in a panel of different tumor cell lines (Fig. 8c). The shielded virus efficiently infected EGFR- and HER2-overexpressing cancer cells, demonstrating again that the shield does not sterically interfere with adapter-mediated receptor binding nor does it prevent virion uptake. In A431 cells the payload expression was slightly reduced upon shielding (Supplementary Fig. 9b). The analysis of cell binding and entry with A488-labeled EGFR-retargeted virus particles suggested a reduction in cell binding of the shielded virions compared to the control virions in this instance (Supplementary Fig. 9c, d). The underlying reasons are so far unknown. Importantly, however, the antibody 9C12 did not affect the payload expression from the shielded virus, in contrast to the non-shielded virus (Supplementary Fig. 9b).

**Fig. 9:**
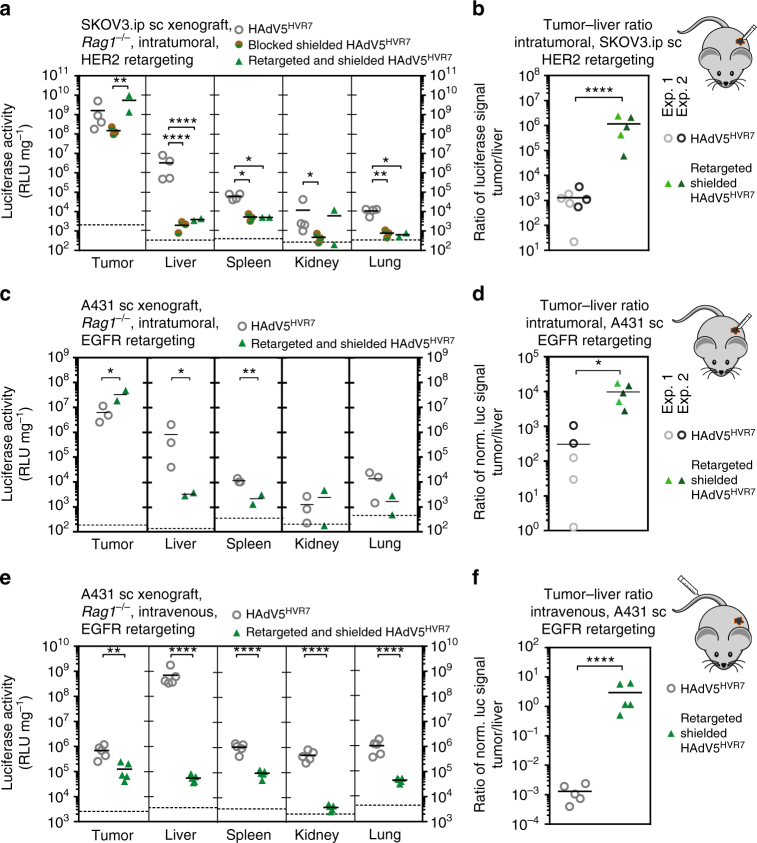
Shielding with retargeting reduces off-targeting and increases tumor–liver ratio. **a** 1.5 × 10^6^ HAdV5^HVR7^ particles were injected into subcutaneous HER2^+^ SKOV3.ip tumors in *Rag1*^-/-^ mice. Gene delivery was analyzed 48 hpi by luciferase activity, normalized to total protein amount. Fiber knob blocking of a shielded virus reduces viral gene delivery to the tumor only slightly compared to virus with free knob. In contrast, HER2 retargeting significantly increases gene delivery within the tumor by a factor of around 40 compared to the fiber knob-blocked virus. Shielding in combination with a blocked fiber knob significantly reduces gene delivery to the liver and all other organs, compared to non-modified virus. Presented data are representative for two independent experiments. **b** Shielding and retargeting significantly increases the tumor-to-liver ratio of gene delivery from ca. 1300 to ca. 1.1-million-fold, an improvement by a factor of ca. 900. Tumor-to-liver ratios of individual mice from two independent experiments are presented. **c** Retargeting of a shielded virus increased viral gene delivery to the tumor after intratumoral injection in A431 xenografts. In addition, off-targeting to liver and spleen was reduced. **d** The ratio of transgene activity between tumor and liver was increased from 300 for the unmodified virus to 9200 for the retargeted and shielded virus. Tumor-to-liver ratios of individual mice from two independent experiments are presented. **e** Intravenous delivery: in comparison to unmodified virus, shielding of an EGFR-targeted virus decreased the gene delivery to the tumor slightly upon intravenous virus injection, but drastically reduced off-targeting to all organs analyzed. **f** Systemic biodistribution was changed by the shield. The tumor–liver ratio in the A431 xenograft was increased by more than 2500-fold (each symbol represents (**a**, **c**, **e**) one organ analyzed with two-way ANOVA of log-transformed data or (**b**, **d**, **f**) the ratio of an individual with two-sided, unpaired Welch’s *t*-test of log-transformed data, *P < 0.05, **P < 0.01, ****P < 0.0001). Background signals from control injections with PBS are indicated by dashed lines

Next, we analyzed the effect of the shield against neutralizing factors other than mAb 9C12. It has been described that preexisting germline IgM antibodies were sufficient to activate the complement cascade and hence inactivate viral particles, especially if FX binding was ablated^19^. Normal mouse serum indeed resulted in a significant decrease of infectivity to 45%, compared to Ig-deficient *Rag1*^-/-^ serum in case of the HAdV5^HVR7^, which was recovered to 84% by the shield (Fig. 8d).

HAdV5 is a common human pathogen with a serum prevalence of 95%^44^. We thus tested the ability of the shield to block neutralization of HAdV5 by human sera. To assess the impact of anti-hexon antibodies on virus transduction, we depleted anti-hexon antibodies by competition with soluble hexon protein. The transduction of the non-shielded HAdV5^HVR7^ is reduced to 38% by human serum, compared to the level after depletion of hexon binders. Strikingly, the infectivity of the shielded virus was not reduced by the hexon binders, indicating that immune-epitopes on the hexon were sufficiently blocked by the shield (Fig. 8e).

### Shield reduces viral gene delivery to liver and spleen

We then studied the effect of the shield on viral biodistribution in an EGFR and HER2 xenograft model in immunodeficient *Rag1*^-/-^ mice. In the subcutaneous SKOV3.ip xenograft, the combination of non-targeted fiber knob blocking and shielding of the virus reduced the gene delivery within the tumor by a factor of 10, compared to unmodified HAdV5^HVR7^ upon intratumoral injection (Fig. 9a). In contrast, the HER2-retargeting adapter significantly increased the tumor-specific gene delivery of the shielded HAdV5^HVR7^ by approximately 40-fold compared to the fiber knob-blocked shielded virus, or 3-fold better than unmodified HAdV5^HVR7^.

Importantly, the shielding and fiber knob blocking, independent of the retargeting DARPin, significantly reduced off-targeting to all other tissues to background levels. The increased gene delivery within the tumor and strong reduction in liver off-targeting led to an improved tumor-to-liver ratio of 1.1 million for the HER2-retargeted, shielded virus compared to a ratio of 1300 for the unmodified HAdV5^HVR7^ upon intratumoral injection (Fig. 9b), and thus about a 1000-fold improvement.

Similar results were achieved in the EGFR-positive subcutaneous A431 xenograft model after intratumoral administration. The combination of shielding and EGFR retargeting resulted in a fivefold increased gene delivery to the tumor (Fig. 9c). At the same time, the off-targeting to the liver and spleen was reduced through the shield by a factor of 260 and 6, respectively. While direct application of HAdV5^HVR7^ to the subcutaneous tumor led to a tumor-to-liver ratio of 300 of the transgene signal, the shielded EGFR-retargeted HAdV5^HVR7^ resulted in 9200 times higher payload activity in the tumor, in both cases upon intratumoral injection (Fig. 9d). Immunohistology confirmed that the shielded and retargeted HAdV5^HVR7^ infects tumor cells in a similar manner to the unshielded, retargeted virion (Supplementary Fig. 3b and Fig. 3).

After intravenous application into tumor-bearing *Rag1*^-/-^ mice, we could confirm the very strong liver tropism of HAdV5^HVR7^ virus (Fig. 9e). Strikingly, a shielded, EGFR-retargeted HAdV5^HVR7^ showed a robust reduction in liver off-targeting by a factor of approximately 14,000. While also the tumor targeting was reduced (×5), the off-targeting to spleen (×11), kidney (×307) and lung (×26) was more strongly decreased. The liver scavenging resulted in a tumor-to-liver ratio of 0.001 for unshielded HAdV5^HVR7^ (Fig. 9f). However, the shielded and EGFR-retargeted virus showed a tumor-to-liver ratio of about 3, constituting an increase by a factor of more than 2500 (Fig. 9e, f). Similar effects were observed in the HER2 xenograft model upon intravenous administration (Supplementary Fig. 10a, b). Here, the liver targeting was massively decreased upon retargeting and shielding, which led to a tumor-to-liver ratio of 2, a 300-fold improvement compared to untargeted and unshielded HAdV5^HVR7^.

## Discussion

Although recent progress in gene therapy clinical trials proved the non-toxicity of adenoviral vectors^45–47^, off-targeting, especially liver sequestration^11^, as well as immune clearance are still major problems for the application of HAdVs. A tumor-to-liver ratio of the delivered viral transgene of only 0.001 illustrates this issue (Figs. 4d and 9f and Supplementary Fig. 10b). Consequently, the primary goal for the future development of such therapies has to be a profound liver detargeting to enable the systemic delivery of tumor-targeted HAdV5 vectors, including a specific targeting of the desired cells.

**Fig. 10:**
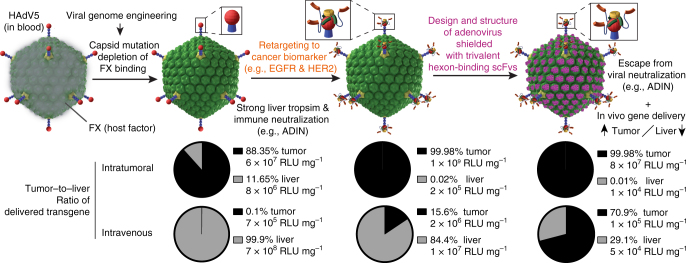
Overview of subsequent modifications for the generation of a new gene vector. Depicted are subsequent steps of engineering. Based on a wild-type HAdV5, which binds FX and e.g. upon intravenous administration this leads to very strong liver tropism. The viral capsid was genetically engineered to ablate the FX interaction. A retargeting adapter allows specific transduction of cancer cells via cell surface markers such as EGFR or HER2. Without the FX coat, the viral capsid can easily be destroyed by the immune system, e.g., by ADIN. We generated a novel artificial shield based on hexon-binding proteins and the viral capsid symmetry determined by EM and crystal structures. The retargeted shielded virus was tested for neutralization, e.g., by ADIN, and for increased gene delivery to the tumor compared to the liver, described as the tumor-to-liver ratio, in both EGFR- and HER2-overexpressing in vivo tumor models

We have developed a viral gene vector with two independent modules, a retargeting adapter^34^ and a scFv shield (reported here), to improve gene delivery to selected tissues (e.g., tumors) in vivo and to reduce liver off-targeting and protect the viral vector from immune neutralization (Fig. 10). Importantly, shield and adapter are separate proteins and can thus be added to virus carrying any payload, without having to reengineer the virus itself.

HAdV5 binds FX, and its interactions make the virus hepatotropic^36^ and activate innate immunity as a PAMP^22^, having motivated the removal of the FX-binding site from the hexon^37^. It has emerged, however, that in the absence of FX HAdV5 remains hepatotropic^19^. Recently, HVRs 1, 2, 5 and 7 have been identified to interact with the Kupffer cell scavenger receptor SRA-II^48^. These observations motivate the creation of a reversible shield around the virion that does not hinder infection nor lead itself to nonspecific binding.

So far, mainly polymeric shielding approaches based on unspecific electrostatic interactions with the virus shell have been reported^49,50^. Covalent coupling of polymers has been attempted as well but found to interfere with intracellular trafficking and uncoating of the capsid^51,52^, which are key to infection^53^. Even if such problems can be solved, the existence of antibodies against polymers like PEG, as recently discussed^54^, might represent another challenge for polymer shields. Another strategy can be protein shields, but so far their affinities were either too weak and could be outcompeted by endogenous proteins^25^ or too strong and they then impaired viral infection^55^. Therefore, we designed a stable, high-affinity multivalent protein shield which binds with an affinity of 10 pM (Fig. 5) and could not be outcompeted by, e.g., the blood-borne FX^25^. Despite this high affinity, we show that the shield does not influence the viral transduction efficiency in vitro (Fig. 8a–b) and in vivo (Fig. 9a–c).

We indeed found that our hexon-specific shield reduced gene delivery to the liver (Fig. 9). Our high-resolution EM and crystal structures reveal that the scFv shield binds to HVRs 2, 5 and 7 of the hexon and, through multimerization, masks the other HVRs, thereby probably blocking the access of scavenger receptors, which are highly expressed on macrophages, present also in lung and spleen. Accordingly, we also observed a reduced viral transgene expression in these organs (Fig. 9a, c, e). The shield might also block a soluble blood factor that binds to the virion and retargets the particle to CAR-expressing tissue. Recently, the presence of such a factor has been described, but not identified, in murine *Rag2*^-/-^ serum^56^. In conclusion, the combination of retargeting and shielding improved the tumor-to-liver ratio upon intratumoral injection from 300 to 7200 for the EGFR^+^ and from 1300 to 1,100,000 for the HER2^+^ xenograft.

Upon systemic administration, the shield and the adapter (providing binding sites for the tumor surface antigens) reduced liver targeting by 14,000-fold (Fig. 9e). However, the virions were not preferentially targeted to the tumor (fivefold reduction), despite showing a gain in specificity and efficacy of tumor targeting upon intratumoral injection (Figs. 3 and 9c). This absence of increase in tumor targeting (Fig. 4) is consistent with a model developed by Wittrup and colleagues^57^, which suggests that for large particles like nanoparticles or viruses, tumor antigen binding will not significantly increase the uptake into the tumor compared to non-targeted particles. In fact, extravasation of large particles is difficult to achieve, as it depends on the fenestration of the tumor, and is limited given by the large size of the viral particles.

We also observed an overall loss of payload signal from the shielded and retargeted virus in all other analyzed organs, including kidney, spleen and lung, upon systemic application, a finding which would be advantageous for tumor applications. At this point, we do not know whether this is due to a decrease of uptake and degradation of the virions in these tissues, or, in cells outside of these tissues, for example, fibroblasts and endothelial cells. Nevertheless, upon intravenous injection, the shield in combination with the adapter increased the tumor-to-liver ratio for the EGFR^+^ tumor model by a factor of 2500, and for the HER2^+^ tumor model 300-fold compared to HAdV5^HVR7^ alone, in both cases resulting in a tumor-to-liver ratio of >2. Future developments will focus on increasing accessibility of the virions to the tumor.

We further analyzed the influence of the shield on neutralization by the immune system. HAdV5 is well controlled by the immune system, being inactivated by the innate^19^ and the adaptive immune system^16^. Here we show that our shield protects the virion from intracellular degradation induced by a monoclonal antibody. The shield also protects from complement-mediated clearance as well as from other, less characterized, hexon-specific neutralization factors in human blood (Fig. 8). Consistently, the hexon has been reported to be the major target of neutralizing antibodies^58,59^, often directed against highly repetitive and charged structures that are, e.g., present in HVRs 1–3 of the hexon^21,60^. The structure of the shielded virion illustrates an extensive coverage of the viral surface, consistent with extensive protection from the immune system (Figs. 6 and 7). Notably, our structure of the Ab-hexon complex also explains the recent finding of an ADIN-escape mutant of HAdV5, carrying the single mutation G443D, selected by directed evolution^61^. We speculate that the insertion of a negative charge in the hexon, facing a cluster of negative charges (Asp32, Asp58 and Asp91) in the light chain of the antibody (Supplementary Fig. 7d), introduces charge repulsions and steric clashes and thus reduces binding of the antibody. To circumvent the high seroprevalence of anti-HAdV5 antibodies, further genetic alterations such as fiber shaft swapping or mutations in the penton base might be envisaged.

Our approach here combines several levels of engineering of different components and improves existing Ad5 vectors. Our strategy to generate mosaic virions is applicable to other serotypes with different features^12,47^. For example, it might be possible to swap the residues in HVRs 2, 5 and 7^58^ forming the scFv epitope onto capsids of other serotypes or chimeric adenoviruses and thereby extend the usage of the available HAdV5 shield.

While the targeting adaptors do not improve net virus localization to the tumor after systemic treatment, they profoundly enhance target-mediated viral infection of the respective cells, demonstrating functionality of the modular adapters in vivo. The adapter is stable in murine serum, an essential feature for in vivo applications. Besides providing new cell surface receptor specificity, the knob-binding part covers a large surface on the fiber knob, and masks the CAR binding site. Similar to others^62,63^, we observed, upon systemic administration, a fiber knob-mediated liver tropism which is reduced by the adapter by approximately 60-fold. This is not dependent on the retargeting moiety per se, but seems to be due to precluding virion binding to CAR. Interestingly, viral targeting to the kidney was also significantly reduced by the knob adapters. So far, the influence of CAR–fiber knob interaction on the liver tropism had only been analyzed in the presence of FX, which in itself contributes to liver tropism^36^. Because of FX protection against neutralization by the innate immune system^19,64^, biodistribution of FX binding-ablated virus particles is best studied in immunodeficient animals.

The generic design of the adapter^34^ allows for the retargeting of virions to various cell surface receptors, as exemplified by HER2 or EGFR in the present study. After intratumoral administration, the tumor-specific viral transgene expression was enhanced through virus retargeting in both HER2^+^ and EGFR^+^ xenograft models by 20- to 34-fold. The retargeting improves the tumor-to-liver ratio from 51 to 7200 and from 1000 to 10,000 for both the EGFR^+^ and HER2^+^ xenograft, respectively, after intratumoral injection. Our cell-specific analysis of gene delivery to the tumor yielded several remarkable findings. First, EGFR retargeting is indispensable to achieve tumor-specific gene delivery in the A431 subcutaneous xenograft, demonstrating the functionality of the adapter. Second, all viruses, independent of retargeting or not, infected murine cells, i.e., fibroblasts in the tumor stroma. We speculate that motifs like the RGD loop in the penton base might play a role, as fibroblasts have been reported to have low CAR but high levels of integrins^65^.

In summary, we describe a proof of principle for a new viral vector using a multidisciplinary approach, combining genetic virus engineering, protein design, cryo-EM, protein crystallography and in vitro and in vivo tumor models to construct a ‘stealth’ tumor-targeting adenovirus, resulting in the first high-affinity yet reversible serum-stable shield described in molecular detail. We improved the protection of the HAdV5^HVR7^ gene delivery vector against immune neutralization and thus improved the tumor–liver ratio of the viral payload. The reduction in viral off-targeting and improved specificity of the payload expression in tumor cells will enable novel concepts for gene delivery of secreted cancer therapeutics with non-replicative viral vectors, which do not rely on transduction of each and every neoplastic cell. Since engineered virions with any payload can be complexed with our shielding and retargeting modules, further engineering can be envisioned to extend the versatility of this modular system.

## Methods

### Virus and cell lines

The human ovarian carcinoma cell line SKOV3.ip was kindly provided by Ellen Vitetta, University of Texas, Dallas, TX. All other cell lines were obtained from the American Type Culture Collection (ATCC; www.atcc.org) and have been verified to be free of mycoplasma contaminations. Two different viruses were used in this project. The replication-deficient HAdV5_ΔE1_GFP, an E1/E3 deletion mutant virus containing the enhanced green fluorescent protein (GFP) gene in the E1 region under the control of the cytomegalovirus major immediate early promoter, was grown in *h*uman *e*mbryonic* r*etinoblast (HER)-911 cells^66^. The replication-deficient HAdV5^HVR7^ is derived from the AdEasy system (Agilent) and contains 4 mutations in the HVR7 (I421G, T423N, E424S and L426Y). The virus was produced in HEK293 cells. Both viruses were purified by a two-step CsCl gradient ultracentrifugation. The antibody 9C12 was obtained from Developmental Studies Hybridoma Bank developed under the auspices of the National Institute of Child Health and Human Development (NICHD) and maintained by the University of Iowa, Department of Biology, Iowa City, IA.

### Purification of retargeting adapters

The DARPin adapters were expressed and purified in *Escherichia coli* BL21^34^. The trivalent adapters were cloned into pQIq vectors containing a N-terminal 3C-cleavable His_10_-tag in *E. coli* XL1 Blue, purified using immobilized-metal ion affinity chromatography (IMAC) with subsequent cleavage of the His-tag. Additional purification steps included anion exchange chromatography using a MonoQ column (GE Healthcare) and size exclusion chromatography (SEC) using a HiLoad 16/600 Superdex 200 pg (GE Healthcare).

### Analysis of viral gene delivery

The retargeting complex was formed by addition of purified adapter in a ratio of 2:1 per virus fiber knob if not stated otherwise. For the shielding, the complex was formed with a 5:1 ratio of trivalent scFv/hexon trimer. The complexes were incubated for 2 h at room temperature (RT). Two different viruses encoding virally encoded reporters, either luciferase or GFP, were used for virus infection assays (see above). 8 × 10^3^ cells were seeded in a 96-well plate 1 day before transduction. For single-cell analysis, expression of the viral transgene GFP upon viral gene delivery was measured by high-throughput microscopy 24 h post infection (hpi) using 8.75 ng virus per well. The cells were fixed with 4% paraformaldehyde (PFA), permeabilized with 0.5% Triton X-100 and stained with 4',6-diamidino-2-phenylindole (DAPI)^32^. DAPI staining was used to mark the cell nucleus, and a custom-made script (Matlab; Mathworks, USA) or a custom-made CellProfiler (version 2.0) pipeline was used to quantify the average nuclear intensity of GFP, and these served as measures of infection efficacy. The raw images, MatLab and CellProfiler routines used in this study will be made available upon request. For luciferase assays, cells were transduced with virus at a concentration of 1000 VP per cell for 4 h until the virus was washed away and replaced with fresh medium. For analysis of viral transgene expression, luciferase activity was determined 48 h post transduction from cell lysate (Luciferase assay system, Promega). For retargeting and shielding, a complex of the adapter and the shield with the virus was formed by mixing the components followed by 2 h of incubation at RT. For the neutralization assays with antibody 9C12, virus was mixed with the antibody (100 ng per mL if not stated otherwise) directly in the well. For complement neutralization, fresh serum from *Rag1*^-/-^ or naive C57BL/6 was obtained and mixed with the virus (2 × 10^8^ VP per10 µL serum) and incubated at 37 °C for 1 h. Subsequently, the mixture was diluted in Dulbecco's modified Eagle's medium (DMEM) with 10% fetal calf serum (FCS) and added to the cells. For neutralization with human serum (Invitrogen), hexon-specific binders were competed by preincubation with hexon (8.8 pmol hexon per 10 µL human serum) at RT for 1 h. All neutralization assays were analyzed as described above for transgene delivery with the retargeting adapters.

For the confocal analysis of cell binding and internalization, cells were seeded on Alcian blue-coated glass coverslips in 24-well dishes (40,000 cells per well) and grown for 2 days. NHS-Alexa-Fluor 488-labeled viruses^67^ (0.23 µg of virus per well, yielding about 10 to 300 bound particles per cell) were bound to cells on ice for 60 min in RPMI-1640 medium (without NaHCO_3_; Sigma) supplemented with 20 mM HEPES and 0.2% bovine serum albumin (BSA) (RPMI-BSA medium). The unbound virus was washed away. For the cell binding analysis, cells were fixed with 4% PFA (pH 7.4), permeabilized with 0.5% Triton X-100, and DAPI stained. Viral internalization was performed by addition of DMEM medium prewarmed to 37 °C and containing 10% FCS. After 1 h at 37 °C, cells were fixed and stained as described above. The samples were imaged with a Leica SP5 confocal laser scanning microscope using a 63× objective (oil immersion; numerical aperture 1.4) and zoom factor 2. Excitations were at 405 nm (DAPI) and 488 nm (Alexa-Fluor-labeled virus). Stacks were recorded at 0.5 µm intervals using 4× averaging and sequential acquisition for the individual channels. Representative images shown in figures represent maximum projections of confocal stacks and were processed with ImageJ, applying the same changes in brightness and contrast to all image groups in the series (http://rsbweb.nih.gov/ij/). All conditions, EGFR retargeting and shielding with retargeting were performed together. For the comparison with the shielded virus, the EGFR targeting of the labeled virus is reused in Supplementary Figure 9.

### In vivo biodistribution of viral transgene

*Rag1*^-/-^ mice were kindly provided by Professor Manfred Kopf, ETH Zürich. The mice were free of all viral, bacterial, and parasitic pathogens listed in the Federation of European Laboratory Animal Associations (FELASA) recommendations. Animals were housed in type III plastic cages (425 × 266 × 150 mm, floor area 820 cm^2^) with autoclaved dust-free wooden bedding (80–90 g/cage) (Schill AG, Muttenz, Switzerland) and autoclaved paper tissues (2/cage) and a paper house as nesting material. They were fed a pelleted mouse diet (Kliba No. 3431, Provimi Kliba, Kaiseraugst, Switzerland) ad libitum and had unrestricted access to sterilized drinking water. The light/dark cycle in the room consisted of 12/12 h with artificial light (40 Lux in the cage) from 0700 to 1900 h. The room temperature was 21 ± 1 °C, with a relative humidity of 50±5% and with 15 complete changes of filtered air per hour (HEPA H 14 filter, Vokes-Air, Uster, Switzerland); the air pressure was controlled at 50 Pa. The studies were approved by the Cantonal Veterinary Office (Zurich, Switzerland). Housing and experimental procedures were in accordance with the Swiss animal protection law and conformed to the European Convention for the protection of vertebrate animals used for experimental and other scientific purposes (Council of Europe no. 123 Strasbourg 1985).

During all in vivo experiments, the experimenter was blinded in regard to the applied treatments. Sample size was determined using G*Power (α: 0.05, β: 0.8) after a pilot experiment. For the xenograft models, 3–6 × 10^6^ tumor cells (SKOV3.ip or A431) were subcutaneously inoculated in 6–8-week-old *Rag1*^*-/-*^ mice. After tumor establishment (approximately 35 days), all mice were grouped in a stratified randomization based on average tumor size. Luciferase-encoding HAdV5^HVR7^ virus particles were injected (for intratumoral administration, 1.5 × 10^10^ VP in 50 µL PBS, for intravenous injection 3 × 10^10^ VP in 100 µL phosphate-buffered saline (PBS)). For retargeted as well as shielded viruses, a complex of virus, retargeting adapter and shield was formed by mixing the components followed by 2 h of incubation at RT as described above. Mice were killed 48 h post injection by CO_2_ and dissected immediately after death. Organs and tumors were harvested. One half of the tumor and left liver lobe as well as the spleen, the left kidney, and the lung were used to measure transgene activity. Luciferase activity was determined from smashed organ samples and normalized to total protein using BCA protein assays. The remaining half of the tumor with surrounding tissue and half of the left liver lobe were fixed in 4% PFA (pH 7.4). According to the 3R principle, the number of mice was kept at a minimum and the retargeting as well as the shielding analysis in vivo was done in parallel. Therefore, both are compared to the same mice which received only HAdV5^HVR7^.

After 24 to 48 h of PFA fixation, the tumor tissue and liver lobe were trimmed and routinely embedded in paraffin wax for histological and immunohistological examinations. Consecutive sections (3–5 µm) were prepared and stained with hematoxylin–eosin or subjected to immunohistology for the demonstration of luciferase, Her2, EGFR, and vimentin (marker of mesenchymal cells). Briefly, after deparaffination, sections underwent an antigen retrieval procedure, achieved by incubation at 98 °C for 20 min in EDTA buffer (pH 9.0; for luciferase) or in citrate buffer (pH 6.0; for HER2, EGFR, and vimentin). This was followed by incubation with the primary antibodies, mouse anti-firefly luciferase (clone Luci17; Abcam; dilution: 1:100) and mouse anti-vimentin (clone Vim 2B4; Dako; dilution: 1:300) for 1 h at RT; rabbit monoclonal anti-EGFR and rabbit anti-HER2 (clone D38B1 (EGFR); dilution: 1:50 and 29B8 (HER2); dilution: 1:400; Cell Signaling) for 15–18 h at 4 °C, blocking of endogenous peroxidase (peroxidase blocking buffer; Dako) for 10 min at RT, and incubation with the detection system (Envision System HPR Mouse and Rabbit, respectively (for luciferase, HER2 and EGFR); DAKO) for 30 min at RT or streptavidin horseradish peroxidase (Dako Real; Dako), with diaminobenzidine as chromogen and counterstaining with hematoxylin.

### Humanization of scFv by CDR grafting

For the CDR grafting of mAb 9C12^35^ onto a stable human framework, the closest germline family was determined^42^. Subsequently, additional stabilizing mutations were introduced (Supplementary Fig. 4a, b). The resulting scFv was expressed in a modified pFL vectors (Geneva Biotech, Switzerland) behind a polyhedrin promoter and a mellitin signal sequence containing a 3C-cleavable His_10_-tag at the C terminus in insect cells using the MultiBAC system^68^. For the trivalent shield, the scFv was fused to one SHP subunit^43^ using flexible glycine–serine linkers (Supplementary Fig. 4c and d). The scFv was purified by IMAC with subsequent cleavage of the His-tag by 3C protease. Further purification steps included cation-exchange chromatography using a MonoS column (GE Healthcare) and SEC using a Superdex 200 (GE Healthcare).

### Affinity measurements based on surface plasmon resonance (SPR)

SPR measurements were performed on a ProteOn XPR36 instrument (BioRad). Hexon protein was purified from HAdV5^HVR7^-infected HEK293 cells with AIEX using DEAE and MonoQ columns (GE Healthcare)^69^ and biotinylated using NHS-biotin (Pierce). The biotinylated hexon was immobilized on a NeutrAvidin NLC sensor chip (BioRad) until 799 resonance units were reached. Purified scFcs (1 to 32 nM) were injected at a flow rate of 60 μL/min for 240 s. Dissociation was followed over at least 600 s. Simple Langmuir kinetic fitting was applied where appropriate (see Results) with the ProteOn Manager software, otherwise a heterogeneous ligand model was used.

### Crystallization and data collection

For the structural analysis of the scFv binding to the hexon, purified hexon (from HAdV5^HVR7^-infected HEK293 cells, using IEX as described above^69^) was mixed with a threefold molar excess of scFv (VL-(G_4_S)_3_-VH) purified as described above, and the complex was purified by SEC in HEPES buffered saline buffer (1 mM HEPES pH 7.4, 150 mM NaCl). The complex was concentrated to 7.5 mg/mL and sparse matrix screens from Hampton Research (Hampton Research, California, USA) and Molecular Dimensions (Molecular Dimensions, Suffolk, UK) were set up to screen for suitable crystallization conditions. Further details about data collection, structure determination and refinement are described in the Supplementary Methods. X-ray data collection and refinement statistics are listed in Supplementary Table 1.

### Single-particle cryo-EM

Vitrified specimens were prepared by pipetting 4 µL of sample on glow-discharged 400 mesh holey carbon R2/1 EM grids (Quantifoil, Jena, Germany) followed by manual plunge-freezing in liquid ethane with blotting times between 1 and 3 s. Image data were recorded using a FEI Titan Krios transmission electron microscope equipped with a Quantum energy filter and a K2-Summit direct electron detector (Gatan, Pleasanton, USA). Further information about the data collection, 3D refinement, and molecular dynamics are described in the Supplement and in Supplementary Fig. 6 and Supplementary Table 2.

### Statistical analysis

Statistical analyses were performed using GraphPad Prism (La Jolla, CA, USA). All data are expressed as means±standard deviation unless indicated otherwise. The statistical analysis for two-group comparisons was performed with Welch’s *t-*test for normally distributed samples with unequal variance. Multiple sample comparisons were analyzed by analysis of variance (ANOVA) followed by post hoc Tukey's test if not stated otherwise. Significance was established at the *P* ≤ 0.05, 0.01, 0.001, and 0.0001 levels, as indicated in the figures.

### Data availability

The raw data of the micrographs are deposited to the Electron Microscopy Public Imaging Archive (public accession code: EMPIAR-10117). The cryo-EM structure of the shielded virus is deposited in the Electron Microscopy Data Bank under the following entry: EMD-3821. The fit derived from MDFF is deposited in the Protein Data Bank (http://www.pdb.org) under the PDB ID: 6EQC and has been linked to EMD-3821. The crystal structure of the hexon–scFv complex is deposited in the Protein Data Bank under the PDB ID: 5OGI. Data supporting the findings of this study are available within the article and its Supplementary Information Files or from the corresponding author upon reasonable request.

## Electronic supplementary material

Supplementary Information
