# Supplementary material for: Adenoviral vector with shield and adapter increases tumor specificity and escapes liver and immune control
Source: Nat Commun. 2018 Jan 31;9:450. doi: 10.1038/s41467-017-02707-6 (PMC5792622; doi:10.1038/s41467-017-02707-6)
Supplement: Supplementary file 1 — Supplementary Information [file 41467_2017_2707_MOESM1_ESM.pdf]

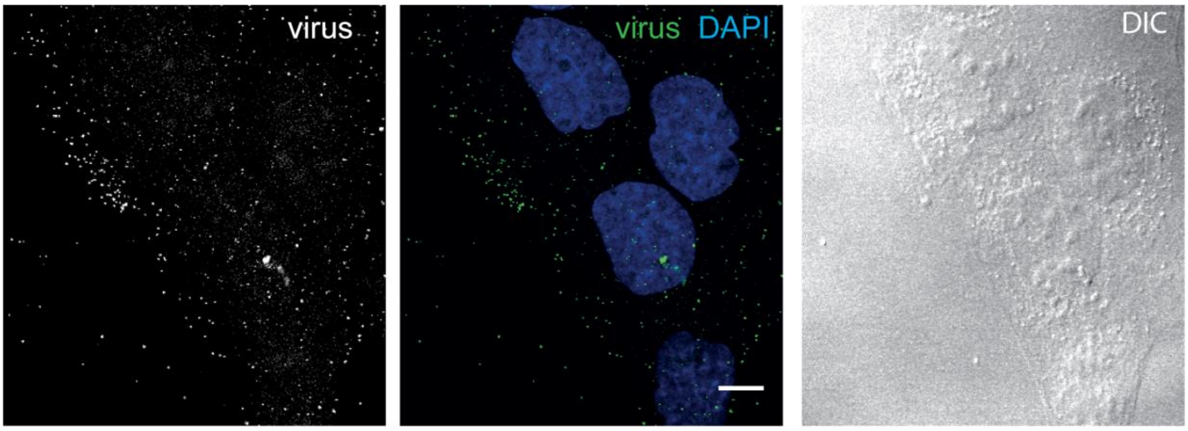

Supplementary Figure 1 | EGFR-retargeted virus binds specifically to A431 tumor cells. In Fig. 1, the differences between unmodified, fiber-blocked and EGFR-retargeted HAdV5^{HVR7} is shown. Only in the case of EGFR-retargeting, massive viral cell binding could be observed. Here, additional examples of the cell-binding of EGFR-retargeted HAdV5^{HVR7} are shown including the differential interference contrast (DIC) which shows the cell outlines. Note the even distribution of Alexa488-labeled virions bound to the cells. A431 cells have high EGFR surface levels and are known to shed the extracellular domain of EGFR, which could explain the occasional virion-binding to cell-free areas.[1]

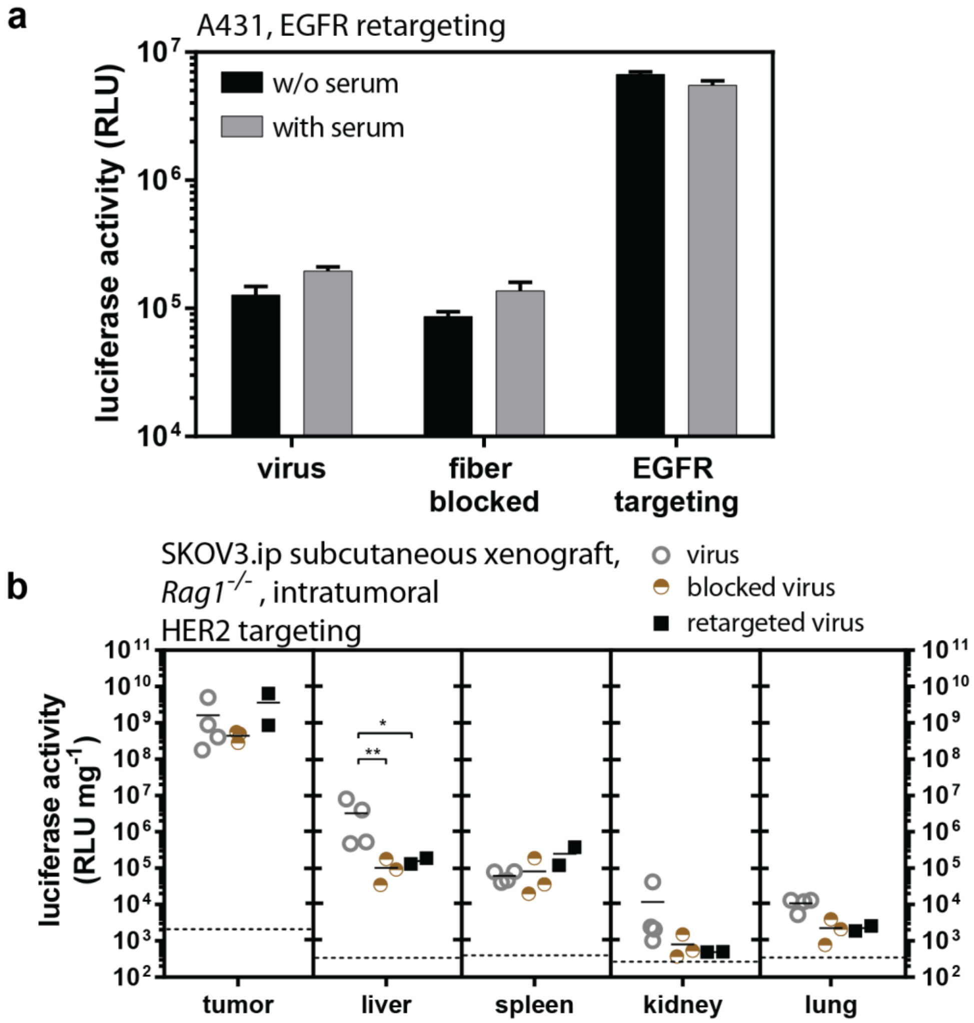

Supplementary Figure 2 | Increase in tumor-specific gene delivery after intratumoral injection of HER2-retargeted HAdV5^{HVR7}. (a) Incubation with Rag1$^{-/-}$ serum had no influence on transduction efficiency of unmodified HAdV5^{HVR7}, fiber knob-blocked HAdV5^{HVR7} or HAdV5^{HVR7} blocked and retargeted to EGFR, irrespective of the targeted receptor, CAR or EGFR. **(b)** Intratumoral injection of HAdV5 with or without HER2-targeting adapter. Transgene expression was higher in the tumor upon HER2 targeting and significantly lower in the liver with both adapters. In all other organs, there was only very low expression of the delivered transgene measured (more than 1000-fold lower than in the tumor). Background signals from control injections with PBS are indicated by dashed lines (each symbol represents one organ, n=2-4, two-way ANOVA of log-transformed data, *P < 0.05, **P < 0.01).

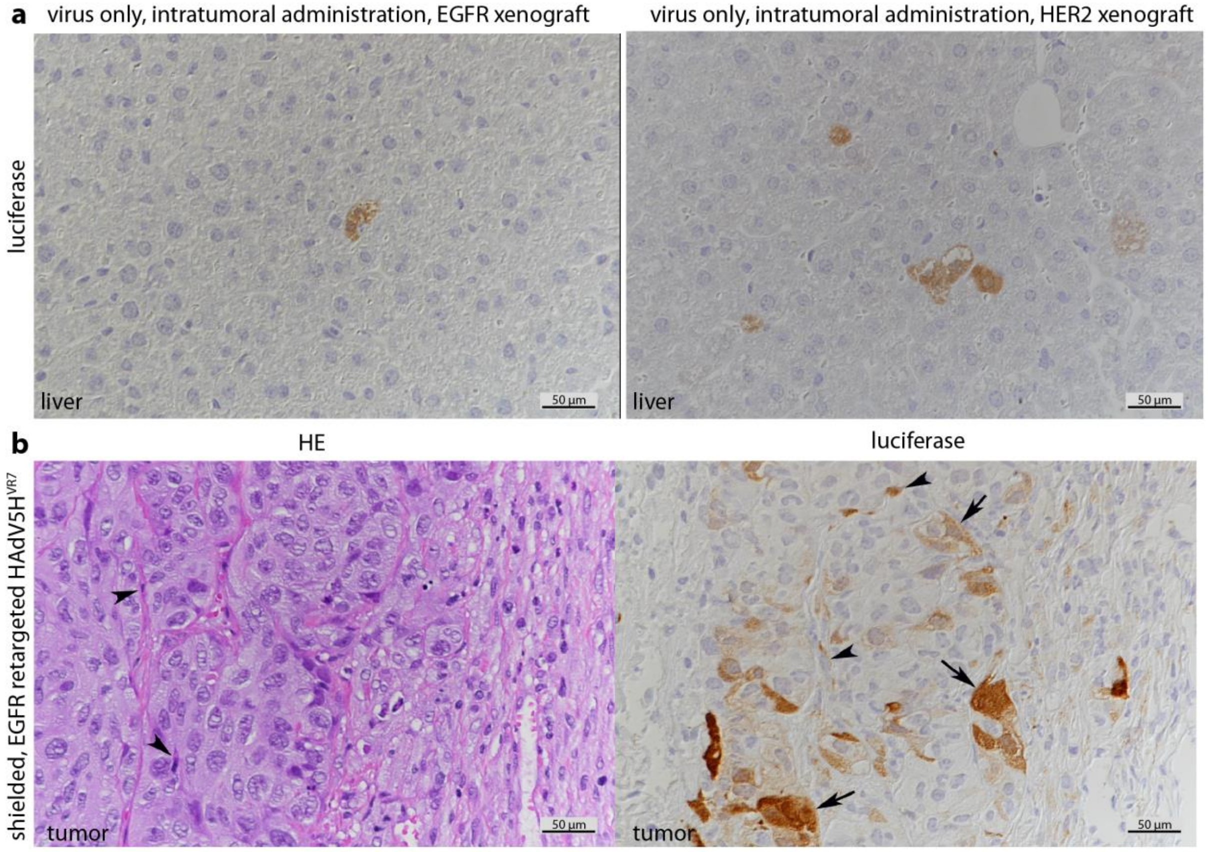

Supplementary Figure 3 | In situ detection of luciferase expression in liver and tumor (a) After intratumoral administration, HAdV5^{HVR7} can escape from the tumor and infect the liver. Luciferase staining confirms viral transgene expression in liver hepatocytes in both the EGFR and HER2 xenograft. Scale bar = 50 µm. **(b)** Shielded and EGFR-retargeted virus is mainly found in viable tumor cells (arrow), although occasional stromal fibroblasts are also luciferase-positive (arrow heads). Analysis of tumor sections were conducted in a blinded fashion, and tumor cells were confirmed by their EGFR expression. Scale bar = 50 µm.

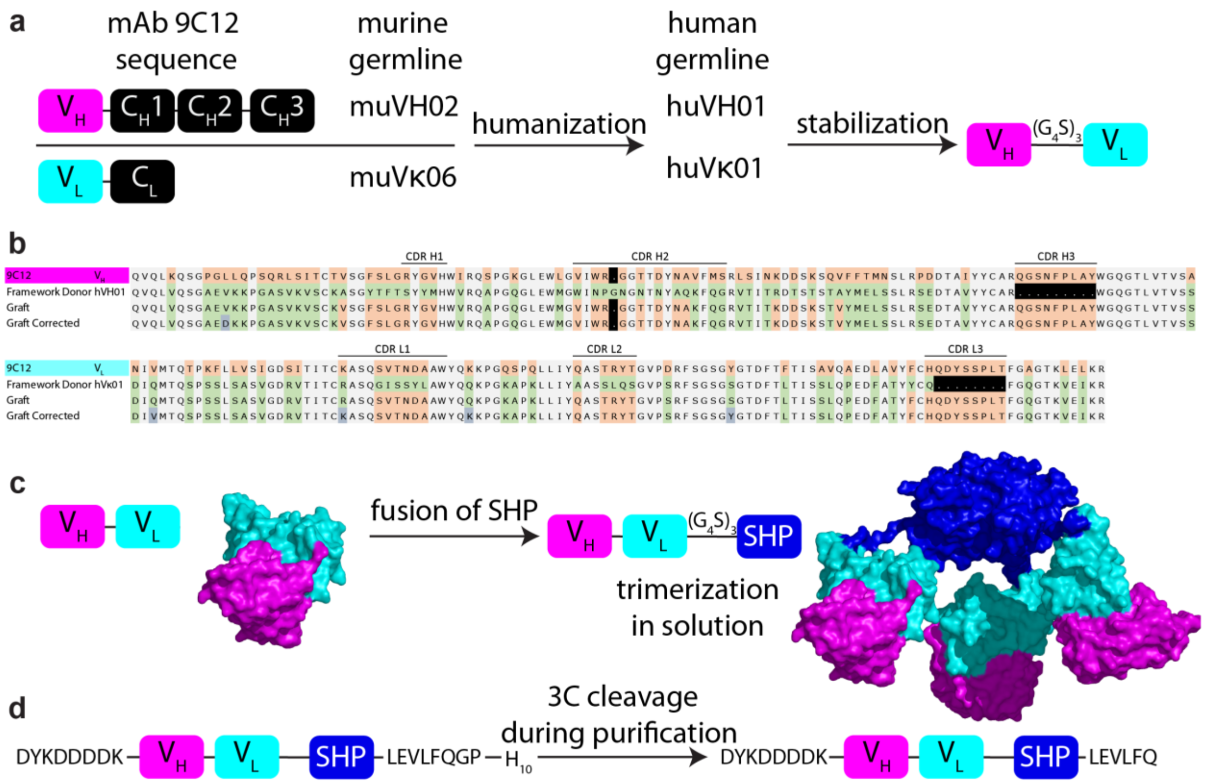

Supplementary Figure 4 | Humanization and trimerization of scFv derived from mAb 9C12 (a) The closest human germline family of the murine mAb 9C12 was identified based on the protein sequence. Further mutations in the framework were included to stabilize the framework.[2] The scFv was linked by a glycine-serine linker of 15 amino acids. **(b)** Sequences of the heavy (upper panel) and light (lower panel) chain. The alignment contains the original mAb 9C12 sequence, the framework donor of the closest human germline, the resulting graft as well as a manually corrected graft for enhanced stability. **(c)** The scFv was fused to the lambdoid phage 21 protein SHP. This protein trimerizes in solution to very stable trimers and thereby forms a trivalent scFv as shown in the structural model. **(d)** The final construct of the trimerizing scFv was expressed in *Sf*9 insect cells with a N-terminal FLAG tag and a 3C-cleavable His_{10} tag. This purification tag was cleaved after IMAC and before cation IEX and SEC.

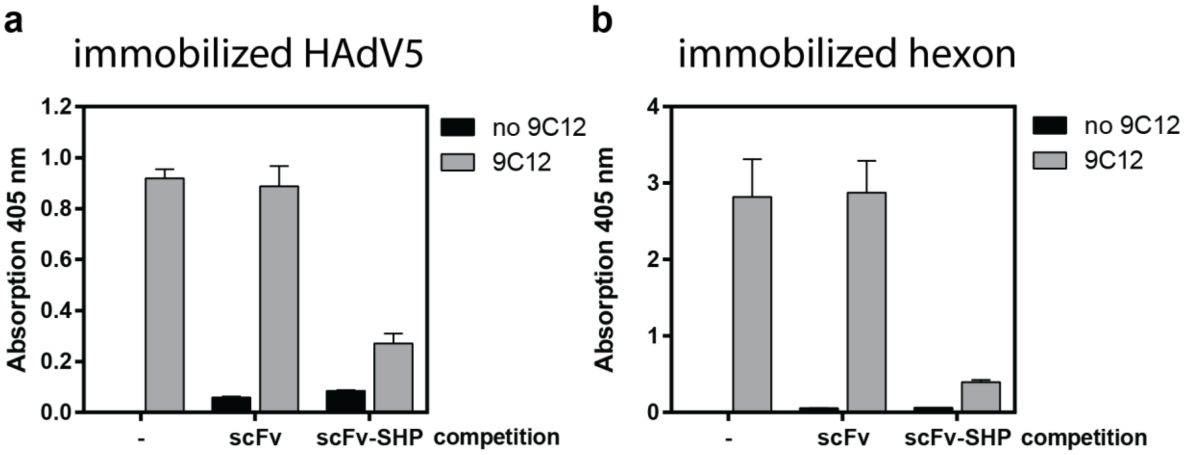

Supplementary Figure 5 | ScFv-SHP blocks binding of an anti-hexon antibody to virus particles and hexon in an ELISA assay. Either complete viral particles **(a)** or purified hexon proteins **(b)** were immobilized on ELISA plates. In a second step, PBS, monovalent scFv or trivalent scFv (termed scFv-SHP) were added in excess to saturate the binding epitopes. After washing steps, binding of the murine anti-hexon antibody 9C12 was measured (gray bars). Background of the secondary anti-mouse IgG detection Ab is shown in black bars. The monovalent scFv could not compete with the bivalent mAb. Only the trivalent scFv-SHP was able to reduce binding of 9C12 to both hexon and compete viral particles.

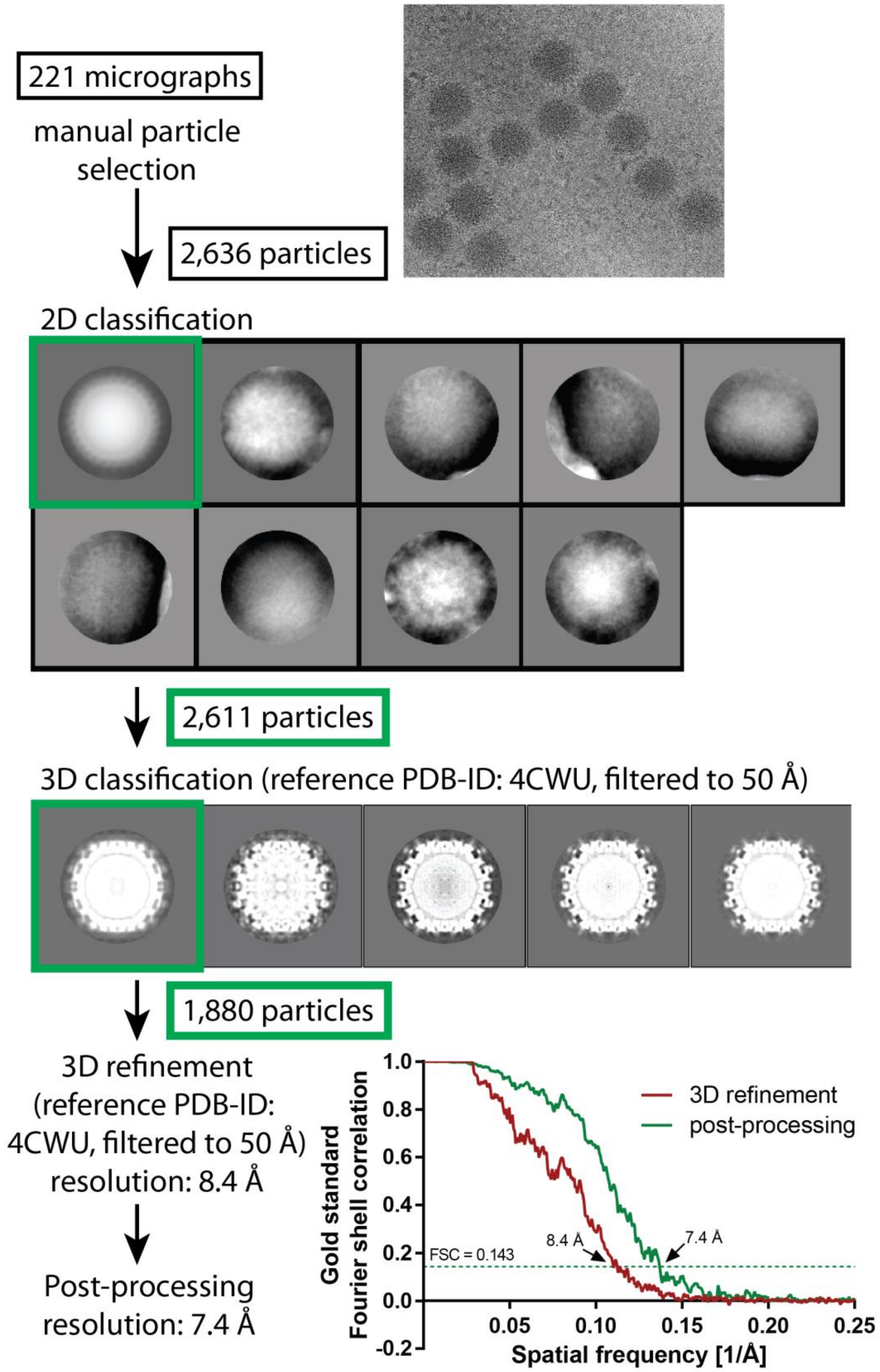

Supplementary Figure 6 | Cryo-EM analysis of shielded adenovirus. The flow chart describes the data processing of the acquired 221 micrographs with the scFv-shielded virus. By manual particle selection, 2,636 particles were used for the 2D classification. The class with the most particles (2,611) was used for a 3D classification with 5 classes and the reference with the PDB-ID 4CWU (filtered to 50 Å). Again, the class containing the most particles (1,880) was used for 3D refinement. Fourier shell correlation (FSC) for the cryo-EM density map was calculated according to the gold standard between independently refined half-maps showing a resolution of 8.4 Å (red FSC curve). Subsequently, relion_postprocessing indicated a higher final resolution of 7.4 Å (green FSC curve). As resolution threshold the FSC = 0.143 criterion was applied.

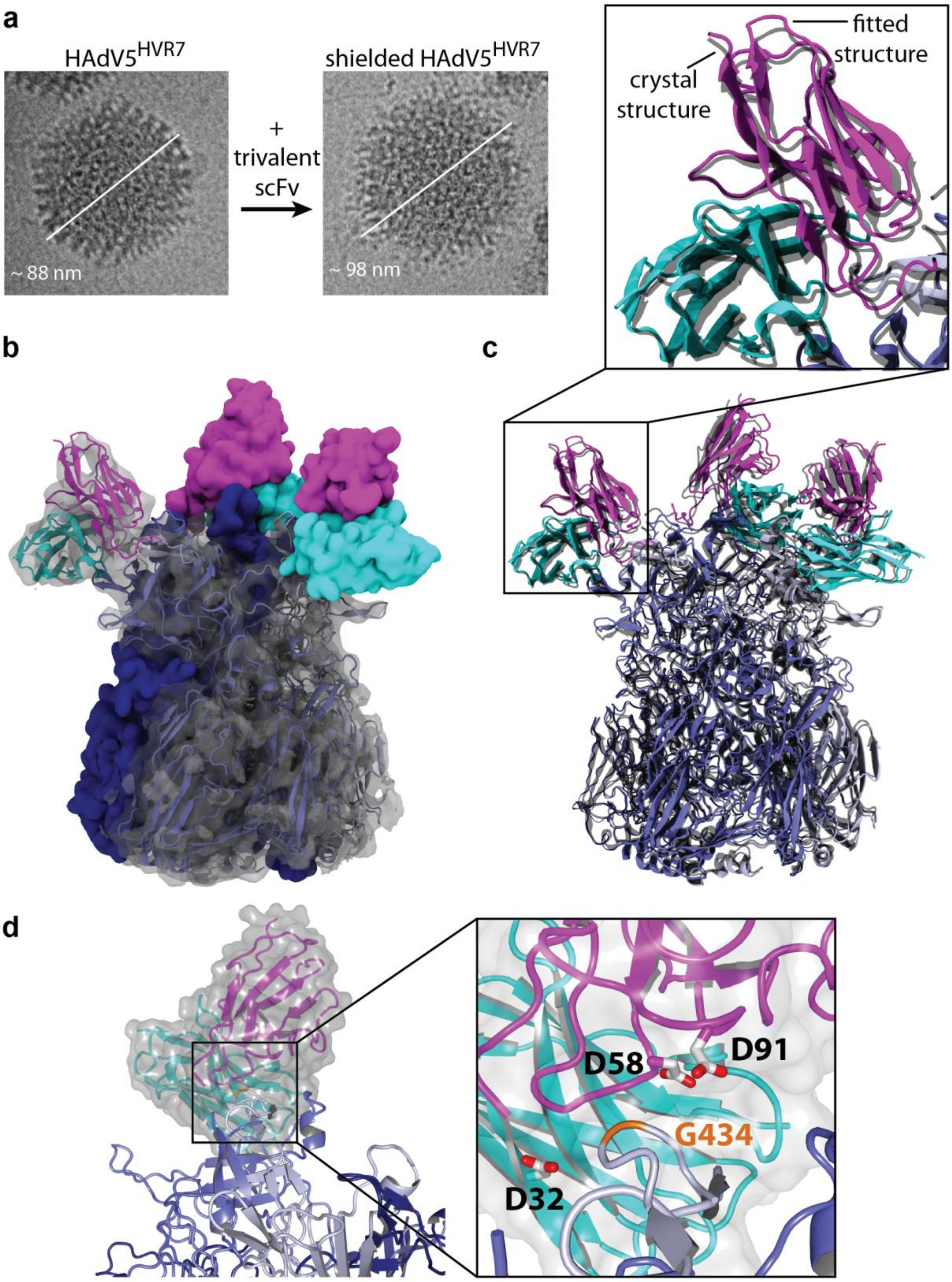

Supplementary Figure 7 |Detailed analysis of the shield by comparison of EM and crystal structure. (a) Exemplary single viral particles with and without shields. The shielded virus appears as a rounder particle and shows an increased diameter of approx. 98 nm, compared to approx. 88 nm for the unshielded virus. **(b)** Molecular dynamics flexible fitting (MDFF) of a scFv model into the EM density. The docked scFv shows a high cross-correlation of 90%. **(c)** Comparison of the crystal structure with MD-simulated docked EM structure. The overall RMSD between both structures is 1.5 Å over 3438 residues. **(d)** Analysis of the epitope of mAb 9C12 explains the impact of the escape mutation glycine 434 to glutamate. The introduction of a negative charge at this position, described by Myers *et al*.[3], results in resistance to 9C12-mediated viral neutralization. Three aspartates located in close proximity on the paratope would cause a charge repulsion in addition to a steric clash.

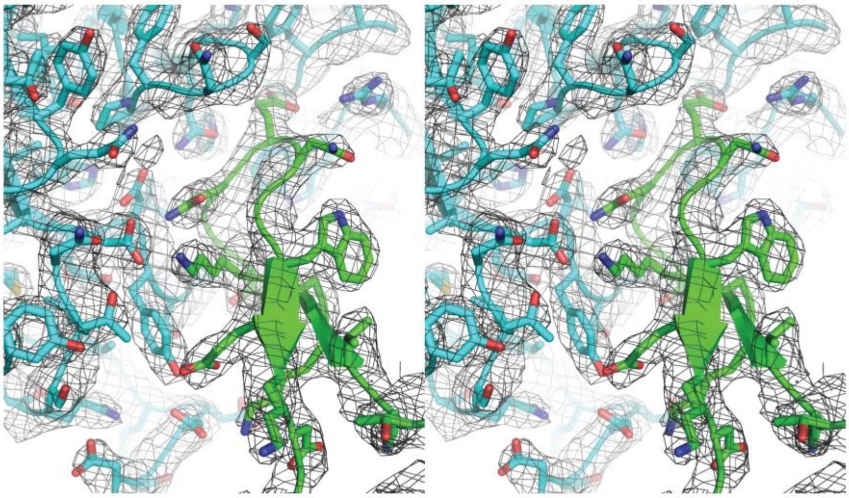

Supplementary Figure 8 | Stereo image of the crystal structure of the hexon-scFv complex. The interface between the scFv (cyan) and the HVR 7 of the hexon (green) is shown as a wall eye stereo image of the 2Fo-Fc electron density map. Density contour level was set to 1.0.

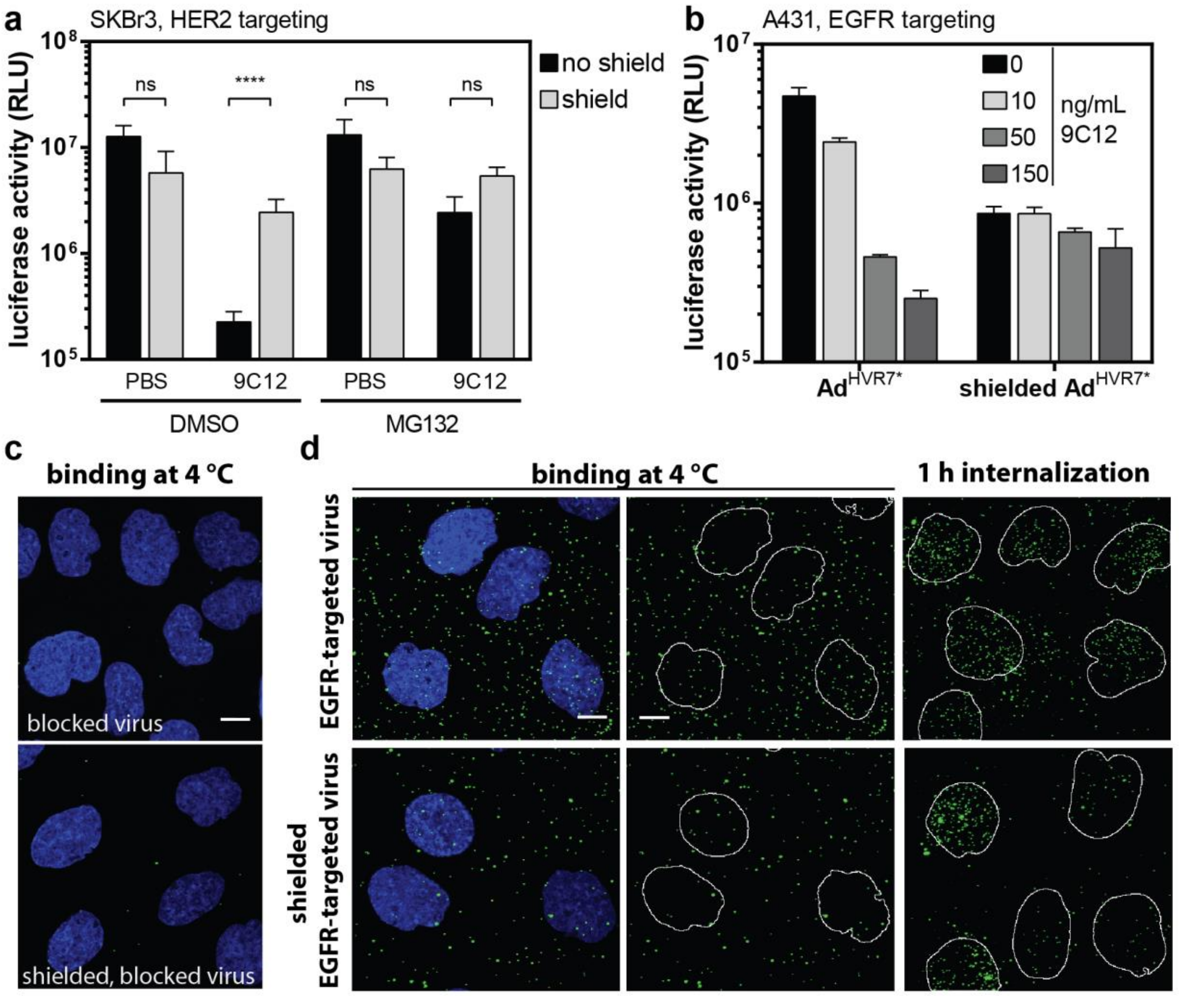

Supplementary Figure 9 | HAdV5^{HVR7} shielded with trivalent scFv overcomes mAb neutralization. (a) ADIN of HER2-targeted HAdV5^{HVR7} can be rescued by the proteasome inhibitor MG132. (mean ± SD, n=4, two-way ANOVA of log-transformed data, ****P < 0.0001) **(b)** 9C12 neutralizes unshielded HAdV5^{HVR7} in a concentration-dependent manner, but virus shielded with the trivalent scFv is protected from 9C12-mediated neutralization. Shielding of the virus, however, reduces the overall transduction efficiency by a factor of 6. **(c-d)** Analysis of shield on virus binding and internalization in A431 cells. Images show maximum projections of confocal sections. **(c)** Both shielded and unshielded viruses with blocked fiber knobs do not bind to A431 cells. The shield therefore does not lead to unspecific cell binding. Scale bar = 10 µm. **(d)** The shielding reduced cell binding of EGFR-targeted particles compared to unshielded HAdV5^{wt} (already shown in Fig. 1, here used for comparison). Upon internalization however, both shielded and unshielded viruses were transported to the nucleus. The shielding thus seemed to have no effect on the intracellular trafficking of the virus. Scale bar = 10 µm.

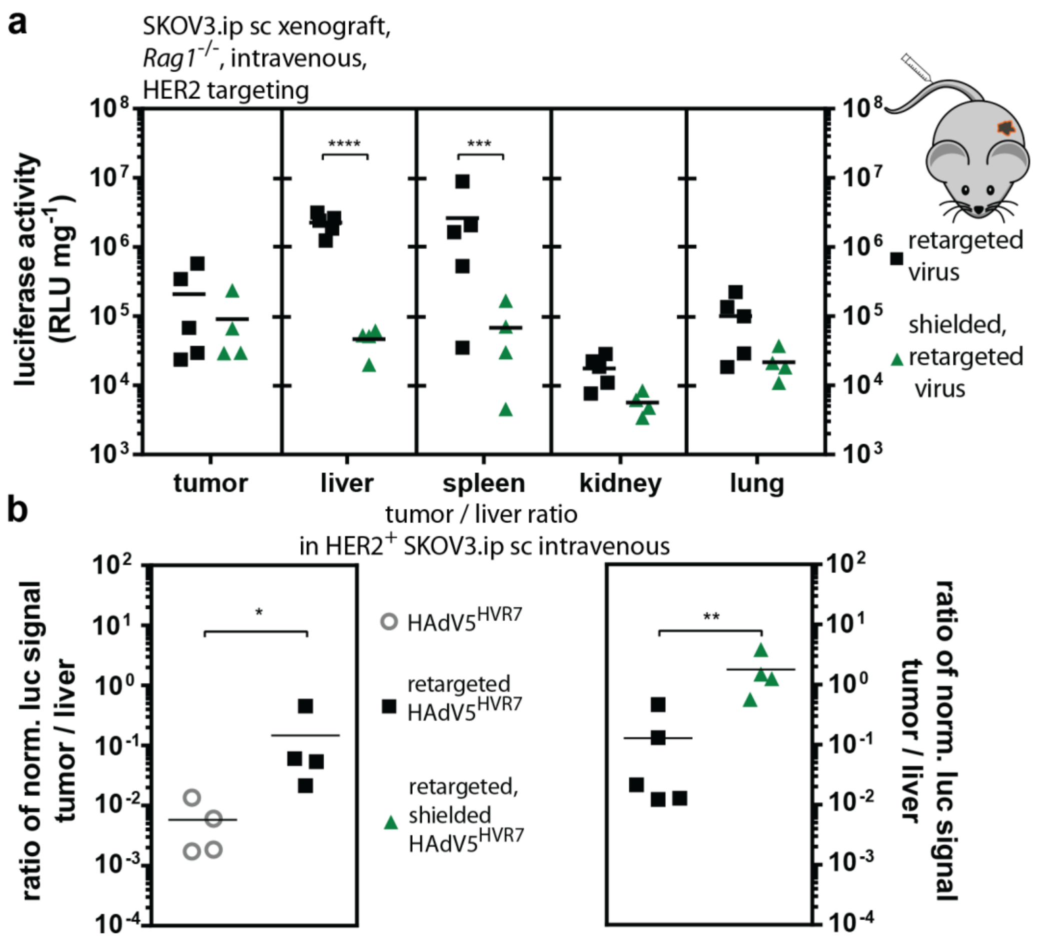

Supplementary Figure 10 | Shielding of retargeted HAdV5^{HVR7} reduces off-targeting and increases tumor-to-liver ratio. (a) 3 × 10^6 HAdV5^{HVR7} particles were injected into *Rag1*$^{-/-}$ mice bearing HER2$^+$ subcutaneous SKOV3.ip xenograft tumors. Gene delivery was analyzed 48 hpi by luciferase activity, normalized to total protein amount. Shielding of a HER2-retargeted virus has no impact of gene delivery to the tumor, but significantly reduces liver and spleen off-targeting by 48- and 39-fold, respectively (each symbol represents one organ, n=4-5, two-way ANOVA of log-transformed data, ***$P < 0.001$, ****$P < 0.0001$) **(b)** Comparison of tumor-to-liver ratio of virus, HER2-retargeted virus and shielded, HER2-retargeted virus in *Rag1*$^{-/-}$ mice with HER2$^+$ SKOV3.ip tumors in two independent experiments with intravenous virus administration. Retargeting and fiber-knob blocking significantly increases the tumor-to-liver ratio from 0.006 to 0.128, a factor of 22. Shielding of the HER2-retargeted virus significantly improves the tumor-to-liver ratio further by 14-fold to 1.8 (each symbol represents the ratio of an individual mouse, two-sided, unpaired Welch's *t*-test of log-transformed data, *$P < 0.05$; **$P < 0.01$).

Supplementary Table 1 | Xray data collection and refinement statistics

	Hexon scFv complex
Data collection	
Space group	$P6_3$
Cell dimensions	
a, *b*, *c* (Å)	158.185, 158.185, 140.805
α, β, γ (°)	90, 90, 120
Resolution (Å)	48.6 - 2.8 (2.9 – 2.8)
R_{meas}	0.427 (5.36)
R_{merge}	0.416 (5.23)
$I / \sigma(I)$	9.76 (0.78)
$CC_{1/2}$	0.99 (0.26)
Completeness (%)	99.4 (99.4)
Redundancy	21.1 (22.0)
Refinement	
Resolution (Å)	48.6 – 2.8
No. reflections	49256 (4905)
R_{work} / R_{free}	0.197/0.237
No. atoms	9624
Protein	9212
Ligand/ion	147
Water	265
B-factors	
Protein	86.01
Ligand/ion	145.23
Water	65.45
R.m.s. deviations	
Bond lengths (Å)	0.010
Bond angles (°)	0.63

One crystal was used for data collection. Values in parentheses are for the highest-resolution shell.

Supplementary Table 2 | CryoEM data collection and refinement statistics

	Hexon scFv complex
Data collection	
Magnification	81,000
Voltage (kV)	300
Electron exposure (e^-/Å^2)	50
Defocus range (µm)	-1 to -2
Pixel size (Å)	0.88
Symmetry imposed	I1
Initial particle images	2,636
Final particle images	1,880
Map resolution (Å)	7.4
FSC=0.143	

SUPPLEMENTARY METHODS

Crystallization and data collection

The protein was mixed with mother liquor in 1:1, 1:2 and 1:5 ratios, dispensed on a Intelli-Plate 96-3 LVR crystallization plate (Art Robbins) with a Phoenix crystallization robot (Art Robbins) and incubated at 4°C with 50 µL reservoir solution. Crystals started growing after 10 days in 1.6 M $(NH_4)_2SO_4$, 10 % dioxane, 0.1 M MES pH 6.5. To harvest crystals for data collection, they were incubated for 10 s in mother liquor containing 20 % ethylene glycol and flash frozen in liquid nitrogen. Data were collected on beamline X06SA at the Swiss Light Source (SLS, PSI, Villingen, Switzerland) with a wavelength of 1 Å using the Eiger16M detector (Dectris, Switzerland). Data processing was done using XDS[4] to a resolution of 2.8 Å, resulting in a CC1/2[5] of 0.26, an I/σ of 0.78 and a completeness of 99.4 % in the highest resolution shell (see Supplementary Table 1).

Structure determination, refinement and analysis

Phases were obtained by molecular replacement using PHASER[6] with the hexon (PDB ID: 3TG7) and a model of the humanized scFv 9C12 as search models. Model building was done using the program COOT[7] and the structures were refined with REFMAC5[8], phenix refine[9] and BUSTER (version 2.10.2. Cambridge, United Kingdom: Global Phasing Ltd). The statistics for the data collection and refinement can be found in Supplementary Table 1. Figures of the structures were generated in PyMOL (Schrödinger, LLC. *The PyMOL Molecular Graphics System, Version 1.8* (2015)).

Data collection and processing of Single Particle Cryo Electron Microscopy

The microscope was operated at 300 keV in zero-loss mode. Using SerialEM[10] movies comprising 50 frames were recorded in superresolution mode with a magnified pixel size of 0.88 Å, a dose rate of 2 e^-/superresolution pixel/s and defocus settings between 1-2 µm underfocus. The frames were recorded with an exposure time of 0.4 s, the total electron dosage of a movie was at around 50 $e^-/Å^2$. The information about data acquisition can be found in Supplementary Table 2. After motion correction of the movies with motioncorr[11] and CTF correction using CTFFIND4[12] image data were further processed using RELION[13]. A total amount of 2,636 particles were manually picked and extracted from the images. The particles were rescaled to 800x800 pixel with a pixel size of 1.96 Å and subjected to a

reference-free 2D classification with 25 iterations and 2,611 particles from one class were further used for the 3D classification. For 3D classification, a 50 Å reference map was generated from a crystal structure of the full adenovirus (PDB-ID: 4CWU)[14] using the e2pdb2mrc.py script from the EMAN2 software[15]. Following 25 iterations of 3D classification with 5 classes and I1 symmetry on a binned data set, 1880 particles from one class were used for the 3D auto-refinement with the same reference and symmetry. Subsequently, the 3D auto-refined structure was sharpened using the relion_postprocessing script. For this, an initial seed mask density threshold of 0.008 was set and the initial mask extended to 2 pixels with a soft edge width of 10 pixels. The gold-standard FSC resolution curve of the final structure before and after the postprocessing is shown in Supplementary Fig. 6. The FSC curve for the postprocessing was generated by RELION whereas the FSC curve for the map of the 3D auto-refinement was generated with the e2proc3d.py script of the EMAN2 software package.

Molecular dynamics

We performed molecular dynamics flexible fitting (MDFF)[16] with NAMD 2.11[17] using the CHARMM36 force field[18], and the system was prepared with the VMD MDFF GUI[19]. In order to get an initial fit we used the Situs package[20] for rigid body docking the crystal structure of the scFv-hexon complex into the map region corresponding to one of the hexons around the 3-fold axis (Fig. 6). The simulation was run with implicit solvent with an ion concentration of 0.1 M. A time step of 1 fs was used. Temperature was kept constant at 310 K with the Langevin thermostat and a damping constant of 5/ps. The structure was coupled to the EM map with a grid scaling of ϕ=0.3. To prevent distortion of the structure, additional restraints were applied in the form of extra bonds to maintain secondary structure integrity and to prevent transitions of *cis/trans* bonds and chirality errors. The cross-correlation between map and structure was measured with the VMD MDFF plugin.

SUPPLEMENTARY REFERENCES

1. Perez-Torres M*, et al.* Shedding of epidermal growth factor receptor is a regulated process that occurs with overexpression in malignant cells. *Exp. Cell Res.* **314**, 2907-2918 (2008).
2. Ewert S*, et al.* Stability improvement of antibodies for extracellular and intracellular applications: CDR grafting to stable frameworks and structure-based framework engineering. *Methods* **34**, 184-199 (2004).
3. Myers ND*, et al.* Directed evolution of mutator adenoviruses resistant to antibody neutralization. *J Virol* **87**, 6047-6050 (2013).
4. Kabsch W. Xds. *Acta Crystallogr. D Biol. Crystallogr.* **66**, 125-132 (2010).
5. Karplus PA, Diederichs K. Linking crystallographic model and data quality. *Science* **336**, 1030-1033 (2012).
6. McCoy AJ. Solving structures of protein complexes by molecular replacement with Phaser. *Acta Crystallogr. D Biol. Crystallogr.* **63**, 32-41 (2007).
7. Emsley P*, et al.* Features and development of Coot. *Acta Crystallogr. D Biol. Crystallogr.* **66**, 486-501 (2010).
8. Murshudov GN*, et al.* Refinement of macromolecular structures by the maximum-likelihood method. *Acta Crystallogr. D Biol. Crystallogr.* **53**, 240-255 (1997).
9. Afonine PV*, et al.* Towards automated crystallographic structure refinement with phenix.refine. *Acta Crystallogr. D Biol. Crystallogr.* **68**, 352-367 (2012).
10. Mastronarde DN. Automated electron microscope tomography using robust prediction of specimen movements. *J. Struct. Biol.* **152**, 36-51 (2005).
11. Li X*, et al.* Electron counting and beam-induced motion correction enable near-atomic-resolution single-particle cryo-EM. *Nat Methods* **10**, 584-590 (2013).
12. Rohou A, Grigorieff N. CTFFIND4: Fast and accurate defocus estimation from electron micrographs. *J. Struct. Biol.* **192**, 216-221 (2015).
13. Scheres SH. RELION: implementation of a Bayesian approach to cryo-EM structure determination. *J. Struct. Biol.* **180**, 519-530 (2012).
14. Reddy VS, Nemerow GR. Structures and organization of adenovirus cement proteins provide insights into the role of capsid maturation in virus entry and infection. *Proc. Natl. Acad. Sci. U. S. A.* **111**, 11715-11720 (2014).
15. Tang G*, et al.* EMAN2: an extensible image processing suite for electron microscopy. *J. Struct. Biol.* **157**, 38-46 (2007).
16. Trabuco LG*, et al.* Flexible fitting of atomic structures into electron microscopy maps using molecular dynamics. *Structure* **16**, 673-683 (2008).
17. Phillips JC*, et al.* Scalable molecular dynamics with NAMD. *J. Comput. Chem.* **26**, 1781-1802 (2005).
18. Brooks BR*, et al.* Charmm - a Program for Macromolecular Energy, Minimization, and Dynamics Calculations. *J. Comput. Chem.* **4**, 187-217 (1983).
19. Humphrey W*, et al.* VMD: visual molecular dynamics. *J. Mol. Graph.* **14**, 33-38, 27-38 (1996).
20. Wriggers W*, et al.* Situs: A package for docking crystal structures into low-resolution maps from electron microscopy. *J. Struct. Biol.* **125**, 185-195 (1999).
